# LAIR1 prevents excess inflammatory tissue damage in *Staphylococcus aureus* skin infection and cutaneous T cell lymphoma

**DOI:** 10.1172/jci.insight.183935

**Published:** 2025-11-13

**Authors:** Hannah K. Dorando, Evan C. Mutic, Kelly L. Tomaszewski, Yulia Korshunova, Ling Tian, Mellisa K. Stefanov, Chaz C. Quinn, Deborah J. Veis, Juliane Bubeck Wardenburg, Amy C. Musiek, Neha Mehta-Shah, Jacqueline E. Payton

**Affiliations:** 1 Department of Pathology and Immunology,; 2 Department of Pediatrics, and; 3 Department of Medicine, Washington University School of Medicine, St. Louis, Missouri, USA.

**Keywords:** Dermatology, Immunology, Oncology, Bacterial infections, Lymphomas, Skin

## Abstract

Patients with cutaneous T cell lymphoma (CTCL) experience high morbidity and mortality due to *S*. *aureus* skin infections and sepsis, but the underlying mechanisms remain unclear. We have previously identified high levels of LAIR2, a decoy protein for the inhibitory receptor LAIR1, in advanced CTCL. Mice lack a LAIR2 homolog, so we used *Lair1-*KO mice to model LAIR2 overexpression. In a model of *S*. *aureus* skin infection, *Lair1*-KO mice had significantly larger abscesses and areas of dermonecrosis compared with WT despite similar bacterial burdens. *Lair1* KO exhibited a pattern of increased inflammatory responses in infection and sterile immune stimulation, with increased production of proinflammatory cytokines and myeloid chemokines, neutrophil ROS, and collagen/extracellular matrix (ECM) pathway proteins, including collagens and complement factors. These findings support the notion that loss of LAIR1 signaling causes an excessive inflammatory response that exacerbates tissue damage and does not improve infection control. Underscoring the clinical relevance of our findings, CTCL skin lesions exhibited similarly increased expression in cytokine and collagen/ECM remodeling pathways, suggesting that high levels of LAIR2 promote excessive inflammatory tissue damage and compromise host defense against *S*. *aureus* infection. LAIR signaling represents a promising target for therapeutic development in CTCL and other inflammatory diseases.

## Abstract 2

We demonstrate that loss of LAIR1 results in inflammatory tissue damage and immune defects that leads to *S. aureus* susceptibility observed in Cutaneous T-cell Lymphoma.

## Graphical abstract

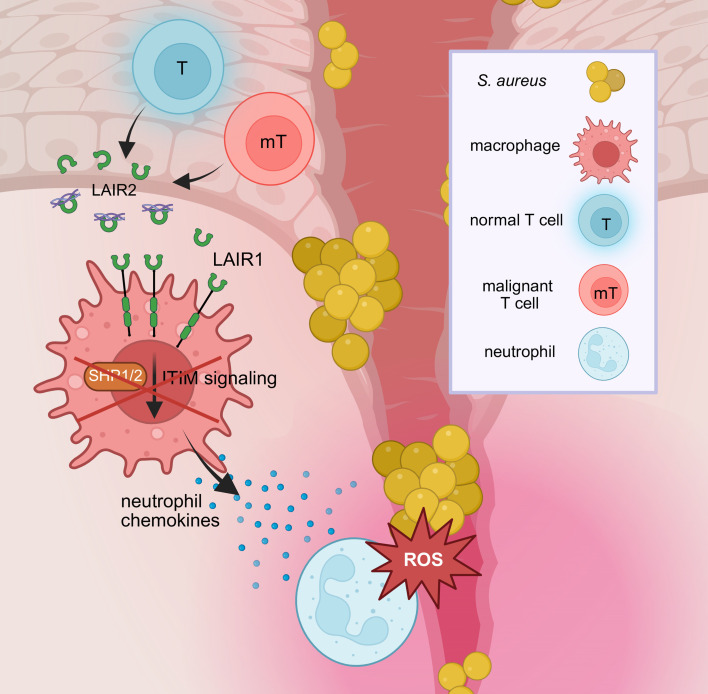

## Introduction

Cutaneous T cell Lymphoma (CTCL) is a cancer of skin-homing T lymphocytes. Patients with late-stage disease have poor survival rates, and the only curative treatment is allogeneic stem cell transplant. Advanced CTCL is marked by inflammatory tissue damage in CTCL-involved skin and immune defects that lead to more severe *Staphylococcus aureus* (*S*. *aureus*) skin and soft tissue infections associated with high morbidity and mortality (1–3). Notably, the underlying mechanisms remain undefined (4–7).

We previously identified aberrant epigenetic regulation and high mRNA and protein expression of leukocyte-associated immunoglobulin-like receptor 2 (LAIR2) in advanced and treatment-resistant CTCL (8). LAIR2 and its paralog, LAIR1, are Ig-like receptors that bind collagens and other proteins with collagen-like domains, including complement pathway proteins such as C1q, which are important in host defense and tissue remodeling (9–12). We and others demonstrated that LAIR1 is highly expressed on macrophages and other innate immune cells; in contrast, LAIR2 is produced primarily by T and NK cells but is secreted into the extracellular space and plasma (8, 13–15). LAIR1 is a transmembrane inhibitory receptor with 2 intracellular ITIM motifs. LAIR2 acts as a decoy receptor, reducing LAIR1 inhibitory signaling and increasing activation signaling in immune cells (10). Upon binding collagen domain-containing ligands, LAIR1 signaling via SHP1 and SHP2 phosphatases negatively regulates many cellular immune activation processes, including cytokine production, calcium signaling, and differentiation (16–20).

Available evidence supports the notion that LAIR proteins likely function in inflammatory responses in infection and inflammatory diseases. Indeed, we and others demonstrated that LAIR1 and LAIR2 expression varies in cell type– and factor-specific patterns in response to pathogen- and host-derived inflammatory factors (13–15). Decreased levels of LAIR1 are associated with rheumatoid arthritis, systemic lupus erythematosus, inflammatory bowel disease, and psoriasis (21, 22). LAIR1 expression in innate immune cells (monocytes, NK cells, neutrophils) may play a modulatory role in immune response to RSV infection (21, 23, 24), severe COVID-19 (25), and pediatric malarial infection (26, 27). These reports support a role for LAIR proteins in inflammatory diseases and immune response to viral and malarial infection, but a role in bacterial infection has not been tested, to our knowledge.

We previously identified LAIR2 upregulation in patients with CTCL, a population with increased susceptibility to invasive *S*. *aureus* infection (1–3). Because mice do not have a LAIR2 gene, *Lair1*-KO mice effectively model loss of LAIR1 inhibitory signaling caused by high levels of LAIR2 (10, 28). We used a well-characterized mouse model of *S*. *aureus* skin infection established by the Bubeck Wardenburg lab in which mice are s.c. inoculated with a high dose of a clinically relevant isolate of methicillin-resistant *S*. *aureus* (MRSA), USA300/LAC (29), causing areas of abscess and dermonecrosis (death of overlying epithelium) that spontaneously resolve within 2 weeks in WT mice (30–32).

The results of in vivo and in vitro studies presented here demonstrate that loss of LAIR1 causes striking differences in the response to *S*. *aureus*. In skin infections, *Lair1-*KO mice developed much larger areas of abscess and dermonecrosis that did not resolve within the experimental timeframe, with no significant difference in bacterial burden. *Lair1-*KO *S*. *aureus* skin lesions also had higher levels of proinflammatory cytokines; these same cytokines were elevated in *Lair1*-KO bone marrow–derived macrophages (BMDMs) infected with *S*. *aureus* in vitro. *Lair1*-KO neutrophils produced higher levels of reactive oxygen species (ROS) yet demonstrated equivalent phagocytosis of *S*. *aureus* compared with WT. In addition, *S*. *aureus* skin lesions from *Lair1* KO exhibited increased expression of genes in extracellular matrix remodeling, collagen biosynthesis and degradation, and complement immune defense pathways. The same collagen/extracellular matrix (ECM) pathways were upregulated in TLR2-activated BMDMs from *Lair1* KO. Comparison of these findings to samples from patients with CTCL with advanced disease revealed that many of the same proinflammatory cytokines and collagen/ECM factors were also significantly elevated in CTCL skin biopsies. Taken together, our findings provide insights into the role of LAIR1 inhibitory signaling as an important modulator of immune response during *S*. *aureus* infection that prevents excess tissue damage. These studies also provide further support for LAIR2’s role in promoting the immune dysfunction and immunopathology observed in CTCL.

## Results

### LAIR2 is elevated in CTCL tumor cells.

We previously reported that high levels of LAIR2 mRNA and protein are associated with advanced and therapy-resistant cutaneous T cell lymphoma (CTCL) (8). LAIR2 is a decoy receptor that disrupts inhibitory signals from its paralog, LAIR1, which normally suppresses inflammatory signaling in immune cells (10). Inflammatory damage to skin and underlying soft tissue, as well as deficits in cellular immunity, are hallmarks of CTCL and are associated with increased incidence of *S*. *aureus* skin and soft tissue infections (1–3, 8). We therefore hypothesized that reduced LAIR1 inhibitory signaling promotes inflammation in CTCL and alters immune response to *S*. *aureus* infection.

**Figure 1:**
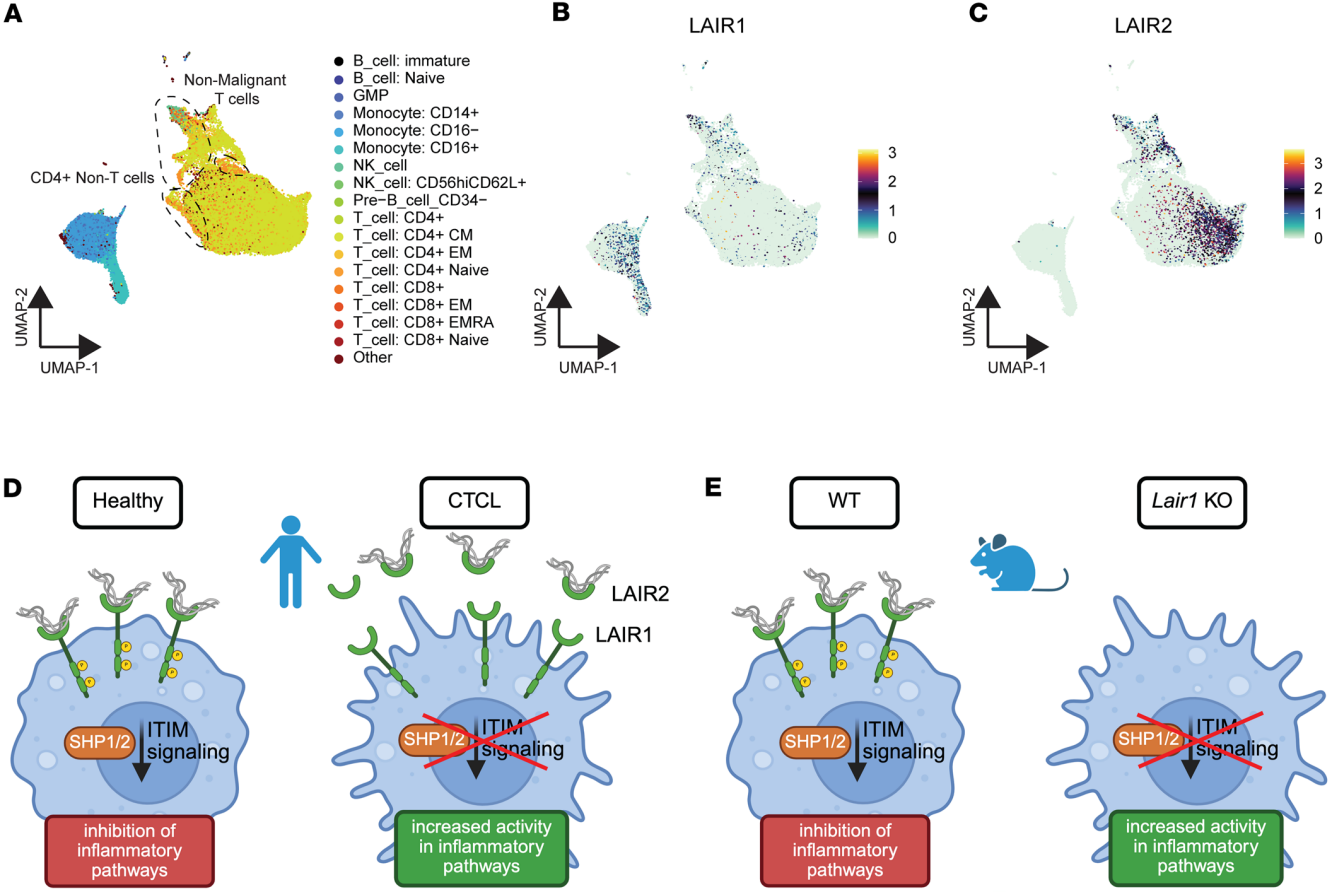
LAIR2 is overexpressed in CTCL and reduces LAIR1 signaling, analogous to *Lair1* KO. (**A**–**C**) CD4^+^ cells were isolated from CTCL patient peripheral blood and analyzed by single cell RNA-Seq. UMAP projections show 92,496 mononuclear cells from 16 peripheral blood samples from 6 patients with CTCL across multiple time points. (**A**) Cell type assignment by highest spearman rho of purified immune populations in the Human Primary Cell Atlas. (**B** and **C**) UMAP plots show expression of LAIR1 (**B**) and LAIR2 (**C**). (**D**) Illustration depicts hypothesis that LAIR2 overexpression reduces LAIR1 signaling in CTCL. (**E**) Illustration depicts mouse model for loss of LAIR1 signaling via KO of *Lair1*.

To begin to test this hypothesis, we first examined single-cell RNA-Seq (scRNA-Seq) from 16 serial peripheral blood samples from patients with CTCL. CD4^+^ cells were enriched from peripheral blood mononuclear cells (PBMCs) to enhance capture of CTCL cells, which are CD4^+^ in the vast majority of patients (33). We used 10x Genomics Chromium Single Cell 5′ Gene Expression and V(D)J Immune Profiling (v1.0) to generate expression and TCR (T cell receptor) repertoire profiles. In total, 92,496 cells passed quality control filters; annotation of cell type was performed using the Human Primary Cell Atlas (HPCA) data set as reference (34). Of 92,496 cells from all samples, the majority were CD4^+^ T cells (76,924); monocytes, which are also CD4^+^, comprised most of the remaining cells (Figure 1A and Supplemental Figure 1A; supplemental material available online with this article; https://doi.org/10.1172/jci.insight.183935DS1). Automated cell annotations were confirmed using canonical marker genes: pan T cell CD3 (CD3D), mature T cell (TRBC2), helper (CD4) versus cytotoxic T cell (CD8A, CD8B), CTCL cell (TOX, KIR3DL2, PLS3), and monocyte (CD14, LYZ, S100A9) (35, 36) (Supplemental Figure 1, B–D). Cell type assignments show a clear separation of CD4^+^ non–T cells, most of which are monocytes, and T cells (Figure 1A). In accordance with our previous work (8, 13), we found that LAIR1 was expressed primarily in myeloid cells (Figure 1B), while LAIR2 is expressed highly in CTCL T cells (Figure 1C). Thus, we hypothesized that LAIR2 secreted from CTCL cells blocks LAIR1 inhibitory signaling in myeloid cells, causing increased inflammation and associated tissue damage, which negatively affect host defense and create a permissive environment for invasive *S*. *aureus* infection (Figure 1D). To test the role of LAIR proteins in *S*. *aureus* skin infection, we used *Lair1-*KO mice to achieve loss of LAIR1 signaling because mice do not have a *LAIR2* homolog (Figure 1E) (10, 28).

### LAIR1 is protective in S. aureus skin infection.

**Figure 2:**
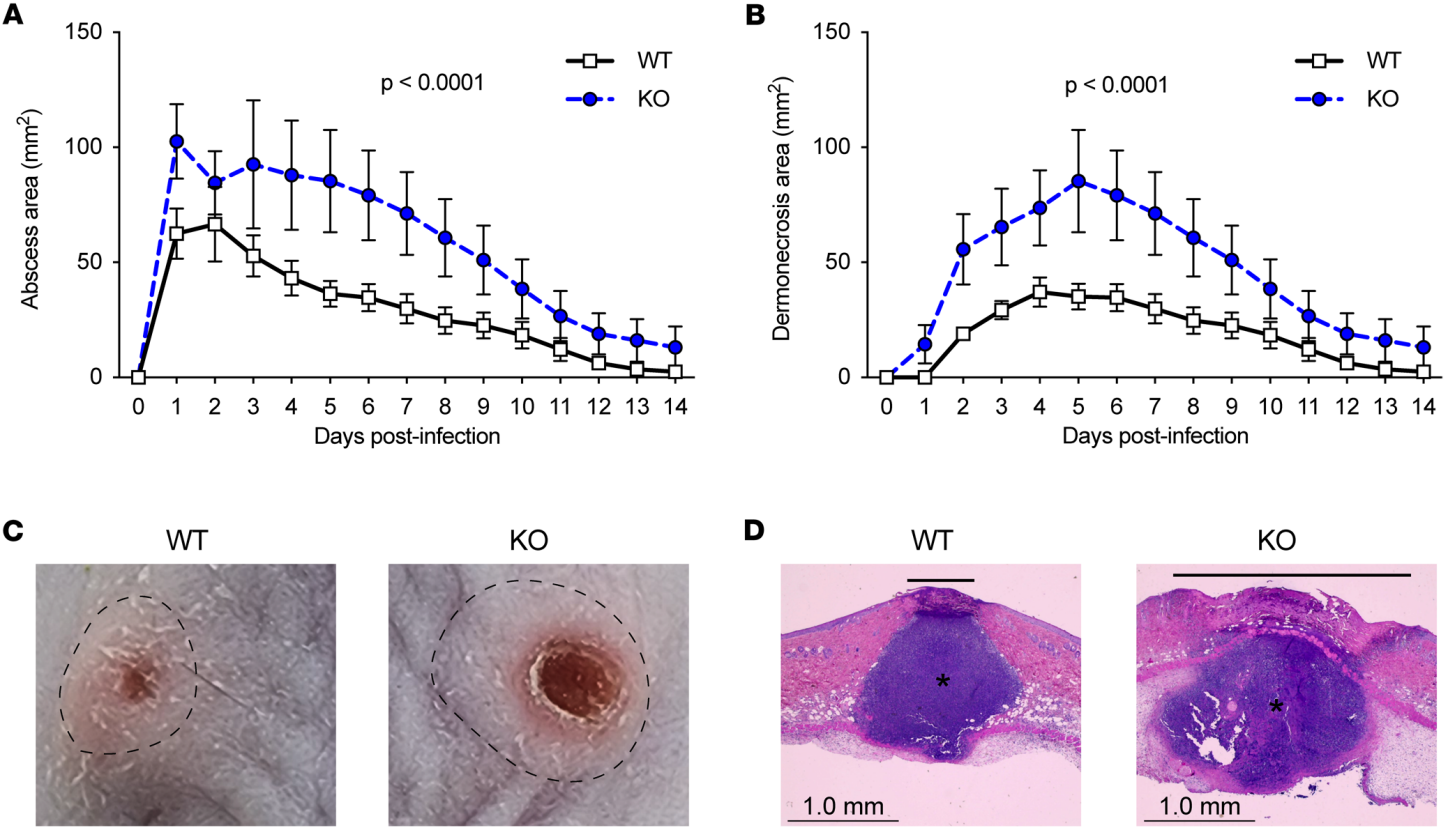
LAIR1 is protective in *S*. *aureus* skin infection. WT and *Lair1-*KO mice were infected s.c. with 1 × 10^7^ CFU *S*. *aureus* USA300 LAC. (**A** and **B**) Lesions were monitored over 14 days and quantified as abscess (**A**) and dermonecrosis (**B**) area (noninvasive measurements, area calculated: A = ([pi/2] × l × w). Statistical analysis by repeated measures 2-way ANOVA with Bonferroni post hoc tests. Results are representative of 5 independent experiments (*n* = 40 WT, *n* = 40 *Lair1* KO). (**C**) Representative gross images of *S*. *aureus* skin lesions in WT (left) and *Lair1*-KO (right) mice on 4dpi. Abscess boundaries are indicated with the dashed lines. Dermonecrosis is the central erythematous lesion. (**D**) Representative H&E stains of lesions at 2 dpi. Scale bars: 1 mm. Asterisks indicate abscesses, and solid lines indicate areas of dermonecrosis, which are larger in *Lair1* KO.

We employed a well-established mouse model of *S*. *aureus* skin infection (30–32) in which WT and *Lair1*-KO mice were inoculated s.c. with 1 × 10^7^ CFU *S*. *aureu*s USA300/LAC. In WT mice, this results in a self-limited skin infection that resolves spontaneously over the course of 2 weeks. The infectious lesions consist of an abscess, which has a collagen-rich capsule generated within 1 day postinfection (dpi) that serves to contain the bacteria, leukocytes, and debris. Dermonecrosis, defined as death of epithelium overlying the abscess, develops at 2 dpi in WT mice. Strikingly, *Lair1*-KO mice develop abscesses that are 2-fold larger on average (Figure 2A) and areas of dermonecrosis that are 2-fold larger and manifest more rapidly (by 1 dpi; Figure 2B) compared with WT mice. Differences in abscess and dermonecrosis sizes were seen beginning at 1 dpi and persisted throughout the experimental timeline, with the greatest differences at 1–8 dpi. This phenotypic difference early in infection suggests a predominant role for myeloid cells. Additionally, while WT mice completely resolve the infection by 14 dpi, an average of over 10% of lesion area remained in *Lair1* KO at 14 dpi. Representative gross images of *S*. *aureus* abscess and dermonecrosis at 4 dpi (Figure 2C) and H&E staining of tissue excised from mice with *S*. *aureus* skin infections at 2 dpi (Figure 2D) demonstrate the larger areas of abscess and dermonecrosis in *Lair1* KO compared with WT. Surprisingly, *S*. *aureus* CFU recovered from skin lesions at 2 dpi or from spleen at 2 or 5 dpi was not statistically different in *Lair1* KO compared with WT (Supplemental Figure 2, A and B). Together, these findings suggest that absence of LAIR1 in *S*. *aureus* skin infection causes greater tissue damage yet does not meaningfully affect bacterial clearance.

α-Hemolysin (Hla) is a pore-forming *S*. *aureus* virulence factor that causes lysis of host cells. Isogenic *hla*-negative *S*. *aureus* strains caused significantly smaller skin lesions with little to no dermonecrosis compared with WT strains (32, 37). To determine whether the larger abscess and dermonecrosis areas in *Lair1*-KO SSTI were dependent on Hla-mediated tissue damage, we used an isogenic *hla*-negative strain of USA300/LAC (Δhla) (37). We inoculated each WT and *Lair1-*KO mouse with USA300/LAC on 1 flank and Δ*hla* USA300/LAC on the opposite flank and compared abscess area (Supplemental Figure 2, C and D) and dermonecrosis area (Supplemental Figure 2, E and F) over 2 weeks. Overall, infection with the Δ*hla* strain reduced the abscess and dermonecrosis sizes in both WT and *Lair1* KO compared with the WT strain. However, Δ*hla* abscesses in *Lair1* KO were larger than WT mice with WT or Δ*hla* infections (Supplemental Figure 2C). Moreover, areas of dermonecrosis in Δ*hla*-infected *Lair1* KO were similar to WT infections in WT mice and were larger than Δ*hla* infections in WT mice (Supplemental Figure 2E).

Statistical analysis of these results confirmed that there are significant differences due to *S*. *aureus* strain and genotype. A 3-way ANOVA was conducted to compare the main effects of genotype and *S*. *aureus* strain as well as their interaction effects on either size of abscess (Supplemental Figure 2D) or dermonecrosis (Supplemental Figure 2F). Effect of genotype (WT vs. *Lair1* KO) was statistically significant for both abscess and dermonecrosis at *P* < 0.0001. Effect of *S*. *aureus* strain (USA300 vs. Δ*hla*) was statistically significant for both abscess (*P* = 0.0008) and dermonecrosis (*P* < 0.0001). The 2-way interaction effect for genotype and *S*. *aureus* strain was not significant for abscess but was significant for dermonecrosis at *P* = 0.0249, indicating that the effect of genotype on dermonecrosis depended on *S*. *aureus* strain. To determine significant differences, 2-way ANOVA with Bonferroni adjustment for α significance level were used as post hoc tests to determine significant differences within the dermonecrosis 2-way interaction effect: dermonecrosis is significantly greater in KO than WT when using USA300 strain (*P* = 0.0004) and showed a nonsignificant trend toward a larger size in KO versus WT when using Δ*hla* strain (*P* = 0.0692). This result was expected due to the overall low amount of dermonecrosis in Δhla infection. Taken together, these data demonstrate that the increased abscess and dermonecrosis sizes in *S*. *aureus*–infected *Lair1-*KO mice were not solely due to the activity of Hla. Therefore, we next sought to define host-derived differences in response to *S*. *aureus* infection.

### Lair1-KO mice produce higher levels of proinflammatory cytokines in skin lesions.

**Figure 3:**
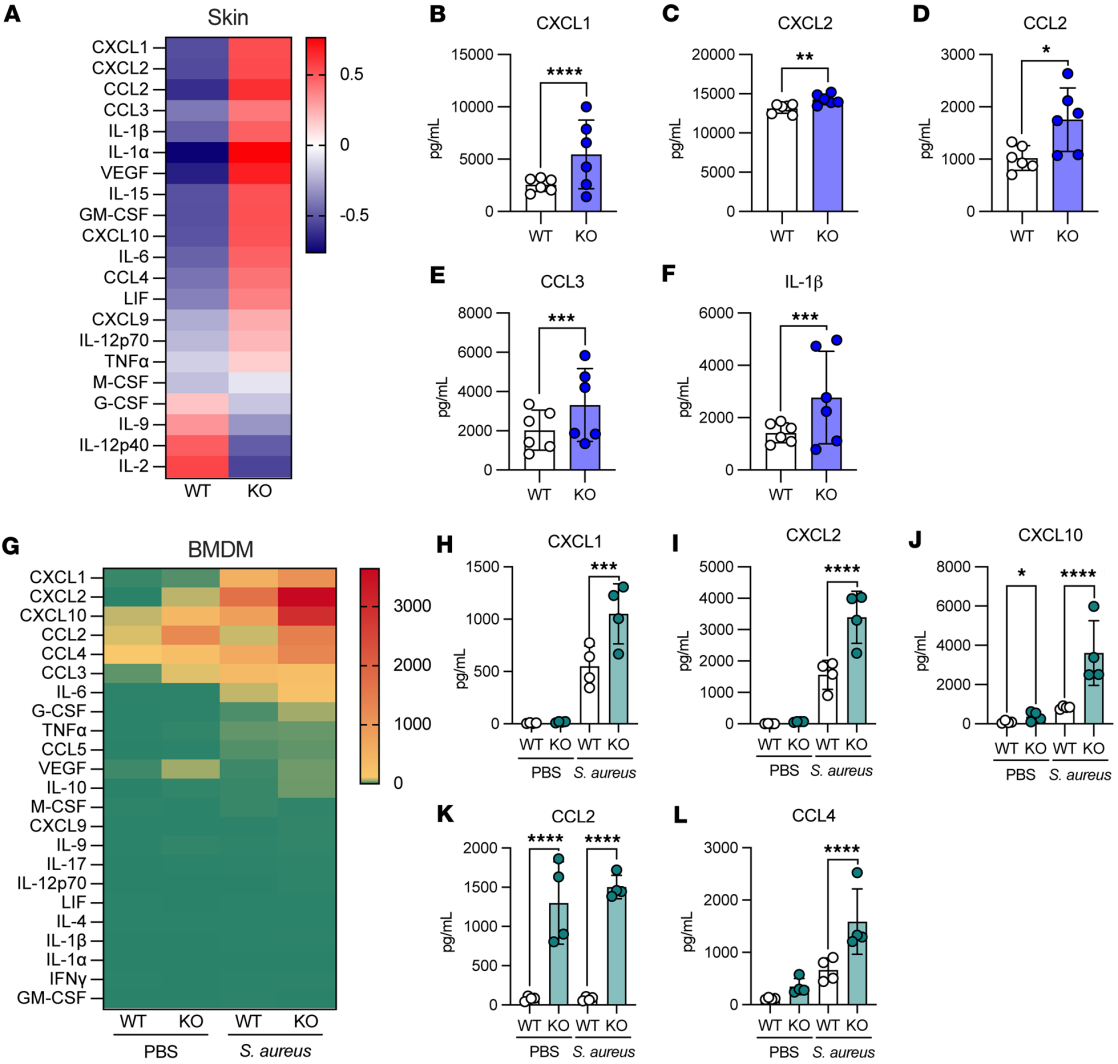
*Lair1*-KO mice produce higher levels of proinflammatory cytokines. (**A**–**F**) Skin lesions were collected at 18 hours after infection, homogenized, and cytokine production measured by multiplex cytokine array. (**A**) Heatmap shows average WT and *Lair1* KO *z*-scored cytokine concentrations. (**B**–**F**) Bar graphs show all cytokines with significantly different expression between WT and *Lair1* KO. (**G**–**L**) BMDMs were generated in vitro and treated with PBS or *S*. *aureus* at a multiplicity of infection (MOI) of 10:1 for 18 hours. Cell supernatant was analyzed by multiplex cytokine array. (**G**) Heatmap shows average WT and *Lair1-*KO cytokine concentrations in PBS and *S*. *aureus* groups. (**H**–**L**) Bar graphs show all cytokines with significantly different expression between WT and *Lair1* KO (*n* = 2 independent infections, 2–6 mice per group). (**B**–**F** and **G**–**L**) Two-way ANOVA with Fisher’s LSD for multiple comparisons. *P < 0.05; **P < 0.01; ***P < 0.001; ****P < 0.0001.

Because we observed larger lesions and more tissue damage in *S*. *aureus* SSTI in *Lair1* KO with no difference in bacterial burden, we hypothesized that *Lair1*-KO mice produce increased proinflammatory cytokines**.** We therefore measured production of 32 cytokines and growth factors from skin punch biopsies from WT and *Lair1*-KO SSTI at 18 hours after infection by multiplex cytokine array. *Lair1* KO skin had an overall pattern of increased proinflammatory cytokine expression (Figure 3A), with higher protein levels of 5 cytokines compared with WT (Figure 3, B–F). Neutrophil-activating chemokines CXCL1 and CXCL2 are both significantly increased, with CXCL1 elevated by more than 100% and CXCL2 elevated by about 8% on average (Figure 3, B and C). Monocyte-activating chemokines CCL2 (MCP1) and CCL3 (MIP1α) were both elevated by greater than 60% on average (Figure 3, D and E). IL-1β, which is classically associated with response to bacterial infection, was increased by over 90% on average (Figure 3F).

Because dermal macrophages express high levels of LAIR1 and are initial sensors of skin infection (14, 38), we hypothesized that macrophages were responsible for the pattern of increased cytokine production in skin infection at 18 hours after infection. To test this hypothesis, we cultured BMDMs from WT and *Lair1*-KO mice and measured cytokine production after PBS treatment or *S*. *aureus* infection using the same cytokine array method. We again observed a significantly increased overall pattern of cytokine expression in *Lair1* KO (Figure 3G), with 5 significantly elevated chemokines, 3 of which were also increased in skin infection: CXCL1, CXCL2, and CCL2 (Figure 3, H–L). CXCL1 and CXCL2 were both increased by over 90% on average (Figure 3, H and I). Strikingly, CCL2 levels in *Lair1* KO were over 10-fold higher than WT in both PBS- and *S*. *aureus*–treated conditions (Figure 3K). CXCL10 was also significantly higher at baseline in *Lair1* KO (Figure 3J). Basal expression of several other chemokines and cytokines, including CCL3, CCL4, CXCL2, and VEGF, were nonsignificantly elevated at baseline in *Lair1* KO, suggesting that loss of LAIR1 releases inhibition of macrophage proinflammatory pathways at rest and may predispose cells to an excessive inflammatory response. Significant increases in proinflammatory cytokines that can recruit and activate multiple immune cell types, including monocytes and macrophages, were detected: CXCL10 was 3-fold elevated (Figure 3J) and CCL4 was elevated by over 130% (Figure 3L) (39, 40). In summary, these data confirm our hypothesis that loss of LAIR1 inhibitory signaling in macrophages results in greater production of proinflammatory cytokines and support the notion that dermal macrophages are at least partially responsible for increased proinflammatory cytokine levels in *Lair1-*KO skin abscess.

### Collagen and ECM remodeling pathways are elevated in Lair1-KO skin abscess and macrophages.

**Figure 4:**
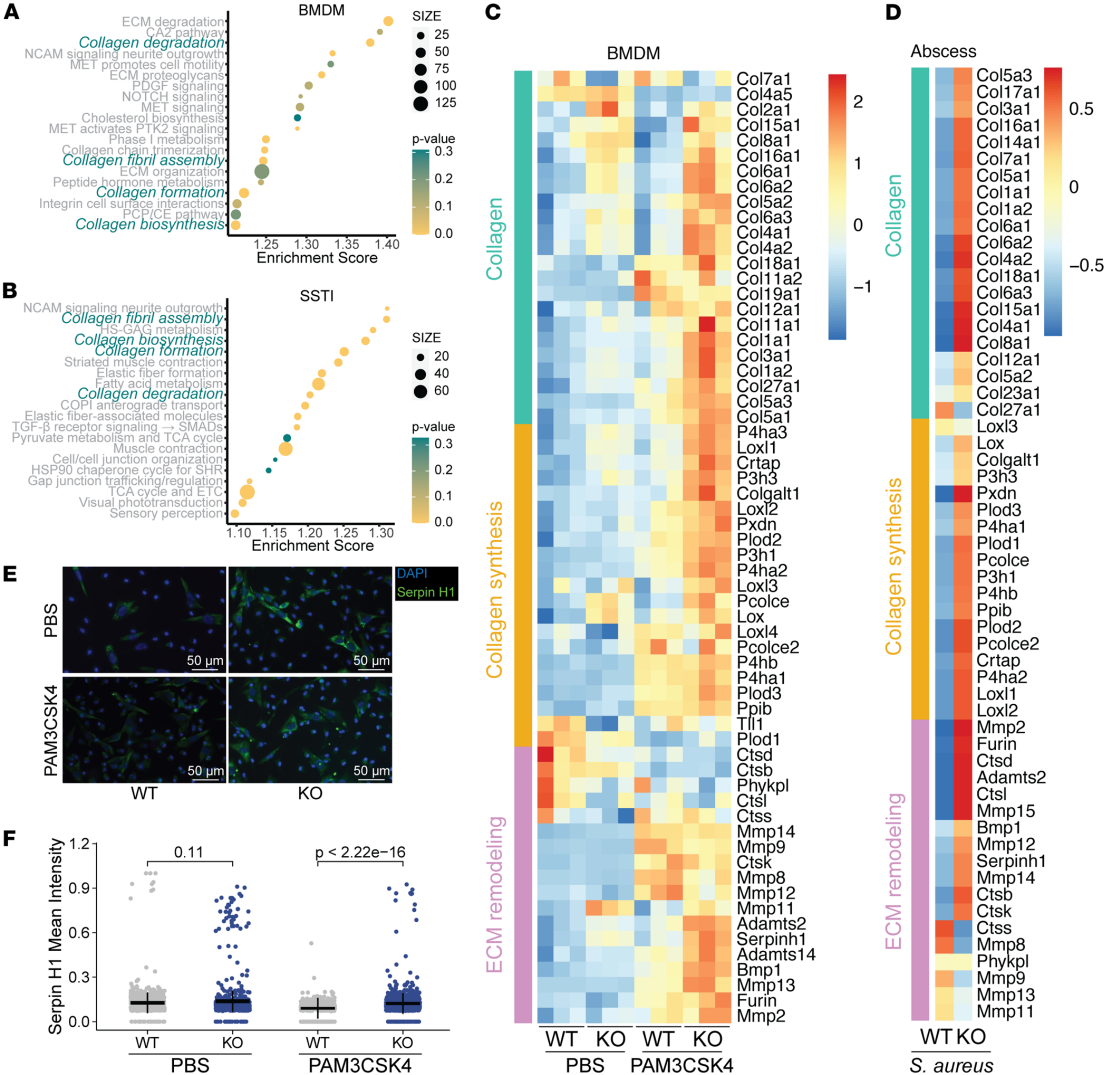
Collagen pathway gene expression is elevated in *Lair1* KO *S*. *aureus*–infected skin and macrophages. (**A** and **C**) BMDM from WT and Lair1-KO mice were treated with PBS or PAM3CSK4 for 6 hours and were then subjected to RNA-Seq. (**B** and **D**) Skin punch biopsies from WT and *Lair1*-KO mouse *S*. *aureus* skin infections were taken at 2 dpi and were then subjected to RNA-Seq. GSEA was used to define the top 20 mouse Reactome pathways with enrichment scores and nominal *P* value in genes upregulated in *Lair1* KO in BMDM treated with PAM3CSK4 (**A**) and 2 dpi SSTI (**B**); collagen-related pathways common to both analyses are bolded in teal. Genes from the 4 pathways, many of which overlapped, were classified into 3 general categories: collagen genes, collagen synthesis genes, and extracellular matrix (ECM) remodeling genes. Heatmaps show *z*-scored expression for these genes for BMDM (**C**) and *S*. *aureus* SSTI (**D**). (**E** and **F**) BMDM from WT and *Lair1*-KO mice were treated with PBS or PAM3CSK4 for 24 hours and were then fixed and stained for Serpin H1. Representative images from 3 independent experiments are shown (**E**). Scale bars: 50 μm. Images were analyzed in CellProfiler and mean Serpin H1 cytoplasmic fluorescence intensity per cell was calculated (**F**). Mean ± SEM are indicated by black bars. Statistical analysis was done by 2-way ANOVA with multiple comparisons.

Our results presented thus far demonstrate that *Lair1* KO macrophages produce high levels of proinflammatory mediators at baseline and in SSTI, leading us to hypothesize that the larger skin lesions and tissue damage in SSTI were due to excess inflammation. To begin to test this, we compared the transcriptomes of WT and *Lair1-*KO *S*. *aureus* SSTI skin biopsies and WT and *Lair1-*KO BMDMs treated with PBS or PAM3CSK4. PAM3CSK4 is a synthetic agonist of TLR2, which is stimulated in infection by bacterial lipoproteins, such as *S*. *aureus* lipotechoic acid, to induce NF-κB pathways (41–43). We defined upregulated genes in RNA-Seq from *Lair1-*KO BMDMs and *Lair1-*KO *S*. *aureus* SSTI compared with WT and used GSEA to identify the top 20 pathways in these genes. Surprisingly, the top 20 Reactome pathways for both BMDMs and skin abscesses included 4 collagen-related extracellular matrix remodeling pathways: “collagen degradation”, “assembly of collagen fibrils and other multimeric structures” (denoted “collagen fibril assembly”), “collagen formation”, and “collagen biosynthesis and modifying enzymes” (denoted “collagen biosynthesis”) (Figure 4, A and B). Notably, there is substantial overlap in the genes in these pathways. Therefore, we combined them into a single set of 98 genes and plotted a heatmap of *z*-scored expression for BMDMs (Figure 4C) and *S*. *aureus* SSTI (Figure 4D) with genes grouped according to function: collagen, collagen synthesis, and ECM remodeling. In BMDMs and SSTI, *Lair1* KO exhibit a clear pattern of increased collagen-associated gene expression in response to PAM3CSK4 and *S*. *aureus*. Several collagen genes are also elevated in *Lair1*-KO BMDMs at baseline (KO + PBS control treatment; Figure 4C), providing further evidence that loss of LAIR1 alters the macrophage phenotype prior to inflammatory insult.

To further support these findings, we verified increased protein levels of collagen pathway genes that had significantly increased mRNA expression in *Lair1*-KO BMDM and skin treated with PAM3CSK4 and *S*. *aureus*. As shown in Figure 4, E and F, we used immunofluorescence to evaluate protein levels of *Serpinh1,* a molecular chaperone that is essential for the proper folding and assembly of collagen. We used CellProfiler to measure average cell fluorescence intensity of Serpin H1 protein in *Lair1*-KO and WT BMDM treated with PAM3CSK4. These studies revealed that Serpin H1 protein expression is 1.35-fold significantly increased (*P* < 2.22 × 10^–16^) in PAM3CSK4-treated *Lair1* KO compared with WT (Figure 4F). In addition, we measured Collagen I by Western blot in BMDM treated with PAM3CSK4, which demonstrated 2.5-fold higher levels in *Lair1*-KO BMDM compared with WT (Supplemental Figure 3, A and B). Consistent with mRNA expression, protein levels of Collagen I are elevated at baseline (PBS control) and with PAM3CSK4 treatment in *Lair1* KO compared with WT. We additionally evaluated thickness of the collagen capsule in frozen sections from WT and *Lair1-*KO skin abscesses stained with Gomori trichrome, which stains collagen fibers (Supplemental Figure 3, C–E). While not statistically significant, there is a trend toward thicker collagen capsule walls in *Lair1* KO; moreover, the overall abscess areas were significantly larger in *Lair1* KO (Figure 1), meaning total abscess wall circumference was correspondingly larger in *Lair1* KO. The larger size would necessitate greater production of collagen and associated ECM remodeling enzymes to achieve, which is consistent with the RNA-Seq, Western blot, and immunofluorescence results. Collagens are also defense molecules, and the increased expression of these pathways may be another facet of the enhanced inflammatory phenotype caused by loss of LAIR inhibitory signaling.

Because LAIR1 also binds collagen domain-containing complement and lectin proteins (10, 15), which are involved in bacterial defense, we next investigated whether these LAIR1 ligands were differentially expressed in *Lair1-*KO PAM3CSK4-treated BMDM and skin abscess. We first determined that *C1qa*, *C1qb*, and *C1qc* were the only members of this group that had detectable expression (averaged normalized counts > 100 for at least 1 group). In BMDMs, *C1qa*, *C1qb*, and *C1qc* exhibited significantly higher expression in *Lair1-*KO cells versus WT when stimulated with PAM3CSK4 (Supplemental Figure 3F). In addition, levels of *C1qa* and *C1qc* in murine skin abscess were significantly higher in *Lair1* KO compared with WT (Supplemental Figure 3G). Together, these findings demonstrate that *Lair1*-KO cells produce higher levels of collagens and collagen defense molecules when infected with *S*. *aureus* or stimulated by TLR2 ligands, providing what we believe to be novel insights into the mechanism by which loss of LAIR1 causes a hyperinflammatory phenotype.

### Lair1 KO produce higher levels of ROS, but phagocytosis of S. aureus is equivalent.

**Figure 5:**
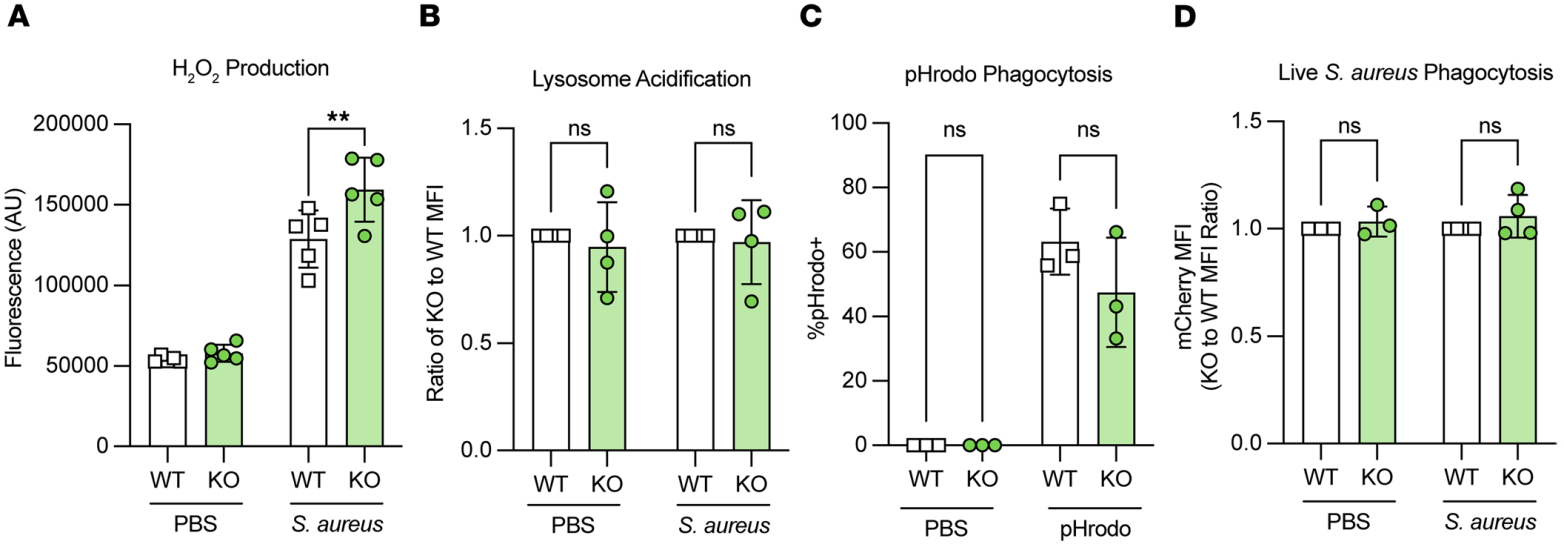
*Lair1*-KO neutrophils produce increased reactive oxygen species but phagocytosis is equivalent to WT. WT and *Lair1-*KO neutrophils were isolated from mouse bone marrow. (**A**) Amplex red fluorescence measured by plate reader indicates hydrogen peroxide (H_2_O_2_) production in neutrophils treated with PBS or *S*. *aureus* at MOI 50:1 for 30 minutes. (**B**) Ratio of WT/KO lysosome probe MFI measured by flow cytometry indicates lysosome acidification in neutrophils treated with PBS or *S*. *aureus* at MOI 100:1 for 30 minutes. (**C**) Neutrophils incubated for 30 minutes with PBS or *S*. *aureus* bioparticles (pHrodo) that fluoresce when acidified in the lysosome were subjected to flow cytometry. (**D**) Neutrophils incubated for 30 minutes with PBS or mCherry-expressing *S*. *aureus* at MOI 50:1 were subjected to flow cytometry. mCherry MFI is shown as a ratio of KO to WT. Statistical analysis for all panels was done by 2-way ANOVA with Fisher’s LSD. **P < 0.01.

We hypothesized that *Lair1*-KO neutrophils may also be hyperactivated in *S*. *aureus* infection because CXCL1 and CXCL2 were elevated in *S*. *aureus* infected skin. To test this hypothesis, we isolated neutrophils from WT and *Lair1-*KO bone marrow, treated them with *S*. *aureus,* and measured ROS production. Compared with WT, *Lair1-*KO neutrophils treated with *S*. *aureus* produced significantly more hydrogen peroxide (Figure 5A), a major component of ROS critical for killing bacteria. Hydrogen peroxide can also cause extensive tissue damage (44, 45). We next assessed intracellular lysosome acidification because it is an important step in bacterial killing, and SHP1 plays a role in regulating this process (46, 47). We incubated neutrophils with *S*. *aureus* and Lys-NIR probe and measured fluorescence by flow cytometry. We did not detect any difference in lysosome acidification between WT and *Lair1-*KO neutrophils (Figure 5B). To determine whether there were differences in phagocytosis between WT and *Lair1-*KO neutrophils, we incubated neutrophils with either *S*. *aureus* bioparticles (Figure 5C) or live mCherry-expressing *S*. *aureus* (Figure 5D); we then measured *S*. *aureus* fluorescence in washed cells by flow cytometry. Neither measure was significantly different in WT and *Lair1-*KO neutrophils.

Monocyte-recruiting and -activating cytokines, particularly CCL2, were also elevated in *Lair1* KO (Figure 3); therefore, we also assessed lysosome acidification and phagocytosis in monocytes. Classical monocytes isolated from WT and *Lair1-*KO bone marrow were assessed using the same methods as for neutrophils (Figure 5). Like neutrophils, monocytes exhibited similar levels of phagocytosis and lysosome acidification in *Lair1* KO and WT (Supplemental Figure 4, A and B). Together, these data demonstrate that *Lair1-*KO neutrophils produce higher levels of ROS in SSTI, but neutrophil and monocyte phagocytosis and lysosome acidification are similar to WT. These data suggest that higher levels of ROS produce excess tissue damage in *Lair1*-KO skin infection yet do not enhance bacterial clearance.

### Cytokines elevated in Lair1 KO are also higher in advanced CTCL.

**Figure 6:**
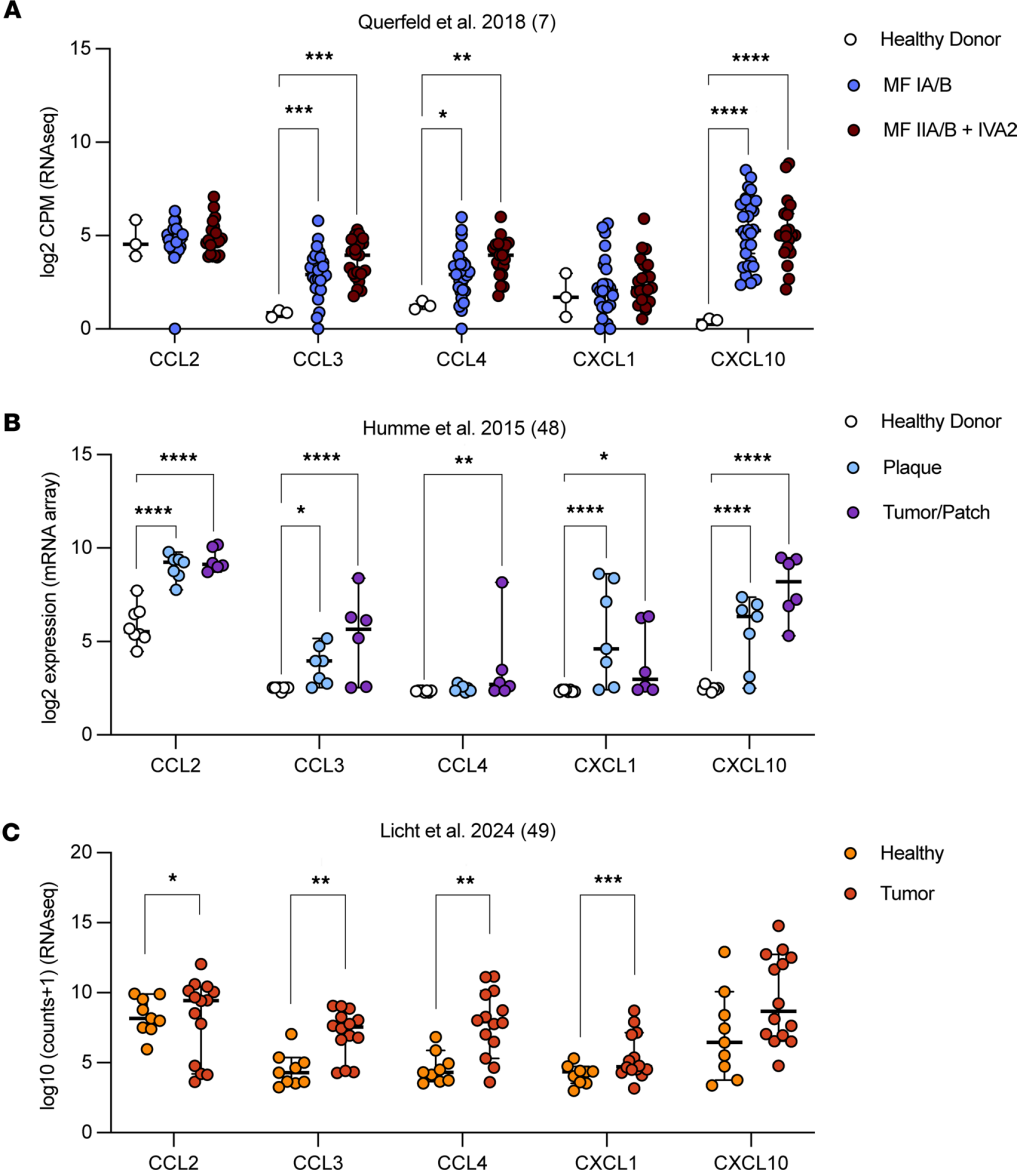
Cytokines elevated in *Lair1* KO are also higher in CTCL. (**A**–**C**) Publicly available CTCL skin biopsy RNA-Seq from ref. 7 (**A**), ref. 48 (**B**), and ref. 49 (**C**) were analyzed using DEseq2. Normalized expression for *CCL2*, *CCL3*, *CCL4, CXCL1*, and *CXCL10* is shown. Statistical analysis for all panels was done by DEseq adjusted *P* value. *P < 0.05; **P < 0.01; ***P < 0.001; ****P < 0.0001.

To determine whether the cytokines that were elevated in *Lair1* KO were also increased in CTCL, we evaluated 3 publicly available RNA-Seq datasets from CTCL lesional skin biopsies (7, 48, 49). We examined cytokine expression for all the significantly increased cytokines in *Lair1*-KO BMDM and SSTI. Skin biopsies were collected from healthy donors and a range of CTCL stages, including: healthy donor, early stage CTCL, and late stage CTCL (Figure 6A) (7); healthy donor, CTCL plaques, or CTCL tumors/patches (Figure 6B) (48); or paired healthy and lesional skin from the same donors (Figure 6C) (49). Notably, none of the biopsies contained active infection. *CXCL1*, *CXCL10*, and *CCL2* were significantly higher in at least 2 of 3 datasets; *CCL3* and *CCL4* were significantly elevated in 3 of 3 datasets evaluated. *CXCL2* and *IL-1b* were not elevated in any of the 3 datasets we evaluated, though other proinflammatory cytokines associated with CTCL pathogenesis, including *CCL5*, *CXCL9*, *IL-15*, *TNF*, and *IFN-g*, were also elevated (Supplemental Figure 5, A–C). Overall, CTCL-involved skin exhibited a pattern of significantly increased expression of cytokines involved in myeloid cell recruitment and activation.

We next evaluated these 3 datasets for expression of the genes in collagen pathways that were elevated in *Lair1-*KO *S*. *aureus*–infected skin and macrophages. Pathway analyses of upregulated genes identified significantly enriched gene sets in immune activation pathways such as CD28 costimulation, IL-3/IL-5/GM-CSF signaling, and IL-10 signaling, though no collagen/ECM remodeling pathways (Supplemental Figure 6, A–C). However, we found that individual genes in these pathways were significantly increased: 5 ECM remodeling enzyme genes were elevated in 3 of 3 CTCL datasets (*CTSS*, *MMP1*, *MMP12*, *CTSB*, and *MMP9*), and 3 collagen genes were elevated in 2 of 3 CTCL datasets (*COL4A4*, *COL6A6*, *COLGALT2*) (Supplemental Figure 6, D–F). These findings are not surprising, given that the biopsies did not involve active bacterial infection or abscess but, rather, ongoing inflammation associated with the cutaneous lymphoma microenvironment (7, 48, 49). In summary, like *Lair1*-KO *S*. *aureus* skin abscess and infected BMDMs, CTCL skin lesions exhibit an overall pattern of increased expression of inflammatory factors, including myeloid cell–recruiting and –activating cytokines and some collagen/ECM remodeling pathway genes.

## Discussion

Our studies presented here reveal roles for LAIR1 inhibitory signaling in CTCL and *S*. *aureus* bacterial infection that, to our knowledge, have not been previously shown. In CTCL, a cutaneous cancer that exhibits striking inflammatory tissue damage, including ulceration, *S*. *aureus* skin and soft tissue infections are common and serious contributors to morbidity and mortality (1–3, 8). We previously identified high levels of LAIR2, a decoy receptor for the immune inhibitor LAIR1, in advanced and therapy-resistant CTCL (8). In studies presented here, we expand upon those findings and demonstrate that loss of LAIR1 signaling causes high levels of proinflammatory cytokines, bacterial defense molecules, and inflammatory tissue damage in *S*. *aureus* infection. Similar elevations were detected in cytokines and complement pathways in CTCL samples. Together, these results demonstrate that LAIR1 is an important regulator of the host immune response and that loss of LAIR1, as in excessive LAIR2 production in CTCL, causes greater inflammation and tissue damage, compromising the host response to *S*. *aureus* infection.

CTCL progression is marked by skin inflammation and barrier breakdown, which culminate in frequent *S*. *aureus* skin and soft tissue infections. LAIR1 had been previously shown to dampen activation in diverse immune cell subsets (9, 12, 18, 50). Based on overexpression of LAIR1 decoy receptor LAIR2 in patients with CTCL with progressive disease (8), we hypothesized that loss of LAIR1 inhibitory signaling may cause increased inflammation in CTCL skin lesions. Inflamed skin has long been recognized as a risk factor for *S*. *aureus* infection, particularly in CTCL (51, 52). We observed a striking phenotype in *Lair1*-KO mice; they suffered larger skin lesions with more tissue damage, as well as higher levels of proinflammatory cytokines and immune defense molecules in *S*. *aureus* infection. It is well established that effective host response to infection requires a fine balance between too little inflammation (inadequate response to control microbial proliferation) and too much inflammation (causing excess damage to host tissues) (53–55). This excess inflammation does not enhance pathogen clearance but, rather, impairs host immune responses and creates opportunities for microbes such as *S*. *aureus* to invade damaged tissues, particularly in CTCL (1, 2, 56, 57). Inhibitory immune pathways, such as LAIR signaling, are an essential component of immune balance and enable nuanced control of immune activation (58). Our results are consistent with this notion and demonstrate that LAIR signaling is an important molecular mechanism underlying the inflammatory tissue damage and increased incidence of *S*. *aureus* infection in CTCL.

LAIR2 is produced primarily by NK and T cells, including CTCL tumor cells, and is secreted, thereby blocking LAIR1 signaling in other cells (8, 10, 59). Innate immune cells, including monocytes and macrophages, express high levels of LAIR1 (13) and, thus, would be affected by LAIR2 blockade. Innate immune cells are the first responders in acute bacterial infection. Therefore, we predicted that reduced inhibitory signaling in these cells due to LAIR1 loss would result in an early and excessive inflammatory response to acute bacterial infection. Consistent with our prediction, our studies here show that *Lair1* KO exhibit increased proinflammatory cytokine levels and larger areas of abscess and dermonecrosis by 1–2 days after *S*. *aureus* infection. While other studies showed an effect of LAIR1 on viral and malarial infection (24, 25, 27), our studies are the first to our knowledge to demonstrate that LAIR1 plays a role in modulating inflammatory response to bacterial infection.

Proinflammatory cytokine production in *S*. *aureus*–infected *Lair1-*KO BMDMs mirrored that of infected skin, suggesting that skin macrophages may be responsible, at least at early infection time points. This notion is further supported by the fact that most are myeloid chemokines, including CXCL1 and CXCL2, which serve to recruit neutrophils from the peripheral blood to the site of infection, resulting in abscess formation (60). Indeed, *Lair1* KO had increased abscess size, likely due to increased neutrophil recruitment and influx. These results are consistent with a study in *Lair1*-KO lung that showed increased neutrophil recruitment upon oropharyngeal administration of CXCL1 and RSV infection compared with WT, although RSV infection did not cause increased cytokine production in *Lair1* KO in this study (61). Our findings thus demonstrate what we believe to be a novel role for LAIR1 in regulating cytokine production and neutrophil recruitment in bacterial infection.

Given these results, we next sought to determine if loss of LAIR1 alters relevant functions of neutrophils or monocytes. ROS production was higher in *Lair1-*KO neutrophils compared with WT, though phagocytic ability in neutrophils and monocytes was equivalent to WT. ROS production causes bacterial as well as host cell death and, if unchecked, leads to substantial tissue injury (62, 63). The larger areas of dermonecrosis in *Lair1* KO may be due, at least in part, to increased ROS production.

Additionally, there are no other studies to our knowledge that demonstrate increased expression of complement, collagens, and ECM remodeling genes in *Lair1-*KO skin infection and BMDMs. Complement proteins are important in host defense against microbial pathogens (64). Collagens and enzymes involved in production and degradation of the ECM are essential for abscess formation (60). Complement component C1q and collagen proteins are ligands of LAIR1. These findings raise the intriguing possibility of a role for LAIR1 in the setting of infection, in which collagen breakdown and complement consumption decrease the pool of LAIR1 ligands, which reduces LAIR1 inhibitory signaling, in turn increasing production of C1q and collagen via a feed-forward mechanism.

Our studies revealed several similarities in CTCL biopsy tissue and *Lair1*-KO *S*. *aureus* infection. Both exhibit increases in macrophage- and monocyte-activating cytokines, including CXCL10, which has previously been linked to early CTCL pathogenesis (65–67). Neutrophil infiltration in CTCL is rare (68, 69), and accordingly, we did not find similar increases in neutrophil activating cytokines in CTCL. Notably, the CTCL skin biopsies evaluated in this study did not harbor active *S*. *aureus* infection. Overall, our results support that inhibition of LAIR1 signaling via LAIR2 overexpression causes macrophage-initiated inflammatory tissue damage and *S*. *aureus* susceptibility in CTCL.

Particularly intriguing are our findings that some proinflammatory cytokines and collagen pathway genes have higher basal expression levels in *Lair1*-KO macrophages. These results suggest that loss of LAIR1 may cause macrophages to be preactivated — i.e., exhibit some characteristics of activation prior to immune stimulus — which could be indicative of altered regulation of immune response at the epigenetic level. These observations require further study to elucidate the underlying mechanisms.

In summary, our studies reveal LAIR1 as a critical modulator of pathogenic inflammation that is important for immune response to *S*. *aureus* infection and protection from CTCL immunopathology. We additionally demonstrate that LAIR1 loss increases the expression of cytokine signaling pathways, which has broader implications for other inflammatory diseases and malignancies in which LAIR1 signaling has been implicated (21, 70–73). Indeed, recent studies in solid tumor models demonstrated reduction in tumor size and increased survival by targeting LAIR signaling using chimeric antigen receptor (CAR) T cells (74, 75). Our findings suggest that LAIR signaling represents a promising target for therapeutic development for CTCL. Future studies are needed to investigate these compelling ideas.

## Methods

### Sex as a biological variable.

For human studies, both male and female patients are included in approximately equal numbers. For animal studies, both male and female mice were used in equal proportions for in vitro experiments, including cytokine analysis from BMDM lysate, RNA-Seq of BMDMs, and experiments involving neutrophil and monocyte isolation. Male and female mice exhibit relevant differences in the stratum corneum — the surface epithelium of the skin. Visualization of epithelial lesions in these models is best performed in male mice as the stratum corneum is several cell layers thicker than in female mice. We confirmed that *S*. *aureus* skin infection in female mice exhibits a similar disease course as in male mice, including differences in *Lair1* KO vs WT. Tissue-based analysis of the clinical correlates of disease are less informative in female mice, due to the very thin stratum corneum. Therefore, to minimize the number of animals needed to obtain consistency in the *S*. *aureus* skin infection models, only male mice were used, which is in agreement with previous reports on this model (76).

### Mice.

WT C57BL/6J and *Lair1*^–/–^ (B6.Cg-Lair1tm1.1Jco/J, JAX Strain #:032788) mice (28) were purchased from The Jackson Laboratory and housed in a pathogen-free environment in the WUSM animal facility. Breeders were fed high-fat chow, and all other mice were fed standard rodent chow (5058; Purina). Inhaled isoflurane was used for all anesthesia.

### Bacteria.

*S*. *aureus* MRSA strain USA300/LAC (gift from Juliane Bubeck Wardenburg lab, WUSM) was used for all infections. Colonies from tryptic soy agar plates were grown overnight at 37°C in tryptic soy broth before being subcultured at a 1:10 dilution and grown until 0.5–0.7 OD_600_. Culture was diluted with tryptic soy broth to OD_600_ 0.48, and bacteria were pelleted, washed in PBS, and diluted to the desired inoculum using the estimated dilution factor 2 × 10^8^ bacteria/mL. Cultures were serially diluted and then plated onto tryptic soy agar to confirm desired inoculum.

### Skin infections.

On day 0, 2- to 3-month-old male mice were anesthetized; then, flanks were shaved and treated with hair removal cream for 2–3 minutes. After 24 hours, flanks were injected s.c. with expected dose of 1 × 10^7^ CFU *S*. *aureus* (actual dose ranged from 1 × 10^7^ to 2 × 10^7^ CFU). Abscess and dermonecrosis length and width were measured over 14 days using digital calipers; then, area of abscess and dermonecrosis was calculated using the formula A = ([pi/2] × l × w). Dedicated mice were sacrificed at 24 or 48 hours after infection for 8 mm skin punch biopsy. For histology, skin punch biopsies were fixed in 10% neutral-buffered formalin for 24 hours at 4°C for paraffin-embedding and H&E staining (Washington University Division of Comparative Medicine Research Animal Diagnostic Laboratory) or fixed for 2 hours in 10% neutral-buffered formalin at room temperature before embedding in OCT Compound (Fisher, 4585) prior to trichrome and H&E staining (WUSM Pulmonary and Critical Care Morphology Core). For CFU analysis or cytokine production analysis, skin punch biopsies were homogenized in PBS and either serially diluted and plated onto tryptic soy agar (31) or stored at –80°C until analysis, respectively. For RNA-Seq, skin punch biopsies were homogenized in TRI reagent (Sigma, 93289) and stored at –80°C until RNA isolation. Results from an entire 8 mm skin punch biopsy are reported for each analysis.

### Preparation of primary cells from mouse bone marrow.

Bone marrow from age- and sex-matched WT and Lair1^–/–^ mice was used for all primary cells. BMDMs were produced by treating bone marrow cells with the equivalent of 10 ng/mL M-CSF from CMG14-12 cells (gift from Deborah Veis lab, Washington University in St. Louis) for 6 days. Primary monocytes and neutrophils were isolated from mouse bone marrow using negative isolation kits (Miltenyi Biotec, 130-100-629 and 130-097-658). Cells were cultured in RPMI 1640 media (monocytes, neutrophils) or α-MEM media (BMDMs) supplemented with 10% FBS, 100 U/mL penicillin (Corning), 100 mg/mL streptomycin (Corning), and 50 μM 2-mercaptoethanol at 37°C in 5% CO_2_.

### Immunofluorescence staining and image analysis.

BMDMs prepared as stated above were plated onto glass coverslips at a density of 3 × 10^5^ cells/mL and incubated for 24 hours with 500 ng/mL PAM3CSK4 (Tocris Bioscience, 4633) or PBS. After 24 hours, cells were fixed in 4% paraformaldehyde for 5 minutes at room temperature and were then stored in PBS until staining. All of the following steps were performed with three 5-minute PBS washes in between: cells were permeabilized with 0.1% TritonX-100 for 10 minutes at room temperature, blocked at 4° C for 24 hours in 2% BSA, incubated with Serpin H1 polyclonal antibody (Invitrogen, PA5-27832) at 1:100 dilution overnight, and finally incubated with Donkey α-Rabbit IgG-Alexa 488 (Invitrogen, A32790) for 1 hour at room temperature in the dark at 1:2,000 dilution. Coverslips were mounted onto slides using Everbrite Mounting Medium with DAPI (Biotium, 23002) and imaged on a Zeiss LSM 880 II FAST confocal microscope. Images were analyzed in CellProfiler (77) in order to quantify expression intensity, with default module settings used except as specified. Nuclei were identified with the IdentifyPrimaryObjects module (using the default Minimum Cross-Entropy thresholding method), cell outlines were identified using the IdentifySecondaryObjects module (using the adaptive threshold strategy, robust background thresholding method, threshold correction factor 1.1, lower threshold bound of 0.07), and cytoplasm was isolated using the IdentifyTertiaryObjects module. Mean fluorescence intensity per cell was exported and analyzed in R.

### Western blotting.

BMDM prepared and treated as for immunofluorescence were lysed in RIPA buffer, heated in Laemmli loading buffer (without 2-mercaptoethanol) at 99°C for 5 minutes, and 40 μg protein per lane loaded on 4%–20% gradient Mini-PROTEAN TGX Precast Gels (BioRad 4561093), transferred to nitrocellulose, blocked with 5% nonfat dried milk in TBS-T, washed, and incubated with Invitrogen Collagen I Polyclonal Antibody (Fisher Scientific, PA126204) 1:500 dilution or Invitrogen GAPDH Polyclonal Antibody (Fisher Scientific, PA1988) 1:4000 dilution before being washed and incubated with anti-rabbit secondary antibody (Invitrogen Goat anti-Rabbit IgG (H+L) Secondary Antibody, HRP, Fisher Scientific, PI32460), prior to incubation with SuperSignal West Pico PLUS Chemiluminescent Substrate (ThermoFisher Scientific, 34577). They were then imaged and bands quantified on an iBright CL1500 Imaging System.

### In vitro functional assays.

Amplex Red Hydrogen Peroxide Assay Kit (Invitrogen, A22188) was used according to manufacturer’s instructions to measure ROS production. To measure phagocytosis and lysosome acidification, neutrophils and monocytes were treated with either pHrodo Green *S*. *aureus* BioParticles (Invitrogen, P35382) or mCherry-expressing *S*. *aureus* (gift from the Veis lab, Washington University) (29) at multiplicity of infection 50:1 with or without lysosome probe NIR (BioLegend, 421916) at a concentration of 1:500. After incubation at 37° C in 5% CO_2_ for 30 minutes, cells were immediately transferred to ice and pHrodo, mCherry, or lysosome probe fluorescence analyzed by flow cytometry.

### Cytokine analysis.

Multiplex cytokine array (32-plex discovery panel, Eve Technologies) was conducted for skin homogenate and BMDM supernatant.

### Patient sample collection.

Deidentified peripheral blood was obtained from patients seen at the Washington University School of Medicine Cutaneous Lymphoma Clinic under IRB-approved protocols with patients providing informed consent.

### Peripheral blood mononuclear cell isolation and scRNA-Seq.

PBMCs were subjected to enrichment and/or depletion using antibody cocktails as detailed below to enable purification of the desired cells. Monocytes and neutrophils were depleted by incubation of peripheral blood samples with RosetteSep Human Monocyte (CD36) Depletion Cocktail (StemCell Technologies, 15628) for 15 minutes at room temperature, layered onto a Histopaque-1077 gradient, and centrifuged at 400*g* for 30 minutes with no brake. The interphase was collected and washed with 10 mL of sort buffer (PBS, 1% FBS, 2 mM EDTA), followed by red blood cell lysis (155 mM NH_4_Cl, 10 mM KHCO_3_, 0.1 mM EDTA) for 10 minutes. For each sample, 10,000–20,000 viable cells were submitted for processing using the 10x Genomics Chromium Controller and the Chromium Single Cell 5′ Library & Gel Bead Kit v2 (PN-1000006), Chromium Single Cell A Chip Kit (PN-1000152), Chromium Single Cell V(D)J Enrichment Kit, Human, Tcell (96rxns) (PN-1000005), and Chromium Single Index Kit T (PN-1000213) following the manufacturer’s protocols. Normalized libraries were sequenced on a NovaSeq6000 S4 Flow Cell using the XP workflow and a 151 × 10 × 10 × 151 sequencing recipe according to manufacturer protocol. A median sequencing depth of 50,000 reads/cell was targeted for each sample. 

### scRNA-Seq data processing and analysis.

Alignment and gene counting were performed using the Cell Ranger pipeline (10x Genomics, v3.0). Genes found in fewer than 15 cells in a given sample were removed. For each patient, gene counts and cells were pooled into a single Seurat (v3.1.4) object (78), and cells containing fewer than 200 or more than 3,000–3,750 expressed genes, more than 8%–10% mitochondrial reads, fewer than 300 or more than 10,000–20,000 UMIs, or classification as a doublet by the R package scDblFinder with parameters dims = 30, clust.method = “fast_greedy” were removed. Normalization and regression of technical variation due to mitochondrial read percentage and read depth was performed with the SCTransform function with variables.features.n = 4,000. Integration to account for experimental variability due to differences in ficoll or buffy coat preparation and batch effects was performed using the Seurat wrapper around the fastMNN function from the batchelor R package (v1.4.0) with n.features = 3,000. Gene expression was normalized and the top 1,500 variable using the “VST” method were calculated. Data were integrated using the harmony (v1.0.0) R package (79) using both patient and batch information to correct for batch effect with up to 10 iterations. The UMAP and neighbors were calculated with Seurat, using 20 dimensions of the harmony calculations. Cell annotation was performed using the singler (v1.4.1) R package (80) using the highest Spearman rho of purified immune populations in the HPCA (34). Cell type designations with less than 50 cells in the entire cohort were reduced to “other.” Automated annotations were checked manually using canonical marker genes. All single-cell visualizations were performed with Seurat and ggplot2 R package (v3.5.1) (81). 

### RNA isolation and RNA-Seq.

RNA was isolated from homogenized mouse skin punch biopsies using a Direct-zol RNA MiniPrep Plus kit (Zymo research, R2072). BMDMs were plated at 2 × 10^5^ cells/mL and treated with PBS or 500 ng/mL PAM3CSK4 (Tocris Bioscience, 4633) for 6 hours. RNA was isolated using an RNeasy Plus Mini kit (Qiagen, 74134). RNA was sequenced by the Genome Technology Access Center at the McDonnell Genome Institute at Washington University School of Medicine on an Illumina NovaSeq-6000 to generate 150 bp paired end reads. RNA-Seq reads were then aligned to the Ensembl release 101 primary assembly with STAR (v 2.7.9a1). Gene counts were derived from the number of uniquely aligned unambiguous reads by Subread:featureCount (v 2.0.32). Isoform expression of known Ensembl transcripts were quantified with Salmon (v 1.5.23).

### RNA-Seq analysis.

R (v 4.1.3) was used for all analyses. DEseq2 (v 1.4.1) (82) was used to normalize gene counts and evaluate significance of individual genes for our own RNA-Seq and publicly obtained datasets. Genes with counts above expression cutoffs (averaged normalized counts > 100 for at least 1 group), significance < 0.5, and absolute log_2_ fold change > 0.1 were analyzed by GSEA (v 4.3.2) (83, 84) desktop software using the MSigDB mouse or human reactome (v.2023.2) gene set for pathway analysis. Heatmaps were generated using pheatmap (v 1.0.12) R package.

### Statistics.

GraphPad Prism 9 (Version 9.4.1) was used for all statistical tests other than RNA-Seq. All *t* tests were 2-tailed and all ANOVA were 2-way. A *P* value less than 0.05 was considered significant.

### Study approval.

Deidentified peripheral blood draws were obtained from patients seen at the Washington University School of Medicine Cutaneous Lymphoma Clinic under IRB-approved protocols with patients providing informed consent. All animal experimental protocols were approved by IACUC of Washington University in St. Louis. All animal experiments were performed in accordance with the *Guide for the Care and Use of Laboratory Animals* (National Academies Press, 2011)s.

### Data availability.

Sequencing data were deposited in GEO under accession nos. GSE269177 and GSE269178. External datasets used include GEO datasets GSE59307, GSE113113, and GSE221148. Additional datasets are available on Zenodo (DOI: 10.5281/zenodo.org/records/17527215). Values for all data points in graphs other than sequencing data are reported in Supporting Data Values file.

## Author contributions

Conception and design were contributed by HKD, DJV, JBW, and JEP. Development of methodology was contributed by HKD, KLT, ECM, and JEP. Acquisition of data was contributed by HKD, KLT, YK, ECM, LT, ACM, NMS, CCQ, MKS, and JEP. Analysis, interpretation of data, and generation of figures were contributed by HKD, KLT, and JEP. Funding acquisition, writing, review, and/or revision of the manuscript were contributed by HKD, ECM, NMS, and JEP. Study supervision was contributed by JEP. All authors reviewed and approved the manuscript.

## Funding support

This work is in part the result of NIH funding and is subject to the NIH Public Access Policy. Through acceptance of this federal funding, the NIH has been given a right to make the work publicly available in PubMed Central.

- American Heart Association Predoctoral Fellowship grant #P23-01044 to HKD.
- Washington University BioSURF funding to ECM.
- Siteman Cancer Center Foundation to NMS and JEP.
- Barnes-Jewish Hospital Foundation Steinbeck Designated Fund to NMS and JEP.
- McDonnell Genome Institute and Illumina research support to NMS and JEP.
- Gateway for Cancer Research Awards to NMS and JEP.
- The Siteman Cancer Center is supported in part by an NCI Cancer Center Support Grant #P30 CA091842.
- ICTS is funded by the National Institutes of Health’s NCATS Clinical and Translational Science Award (CTSA) program grant #UL1 TR00234.

## Supplementary Material

Supplemental data

Unedited blot and gel images

Supporting data values

## Version 1 11/13/2025

In-Press Preview

## Version 2 01/09/2026

Electronic publication
