# Supplementary material for: LAIR1 prevents excess inflammatory tissue damage in Staphylococcus aureus skin infection and cutaneous T cell lymphoma
Source: JCI Insight. 2025 Nov 13;11(1):e183935. doi: 10.1172/jci.insight.183935 (PMC12890506; doi:10.1172/jci.insight.183935)
Supplement: Supplemental data [file jciinsight-11-183935-s249.pdf]

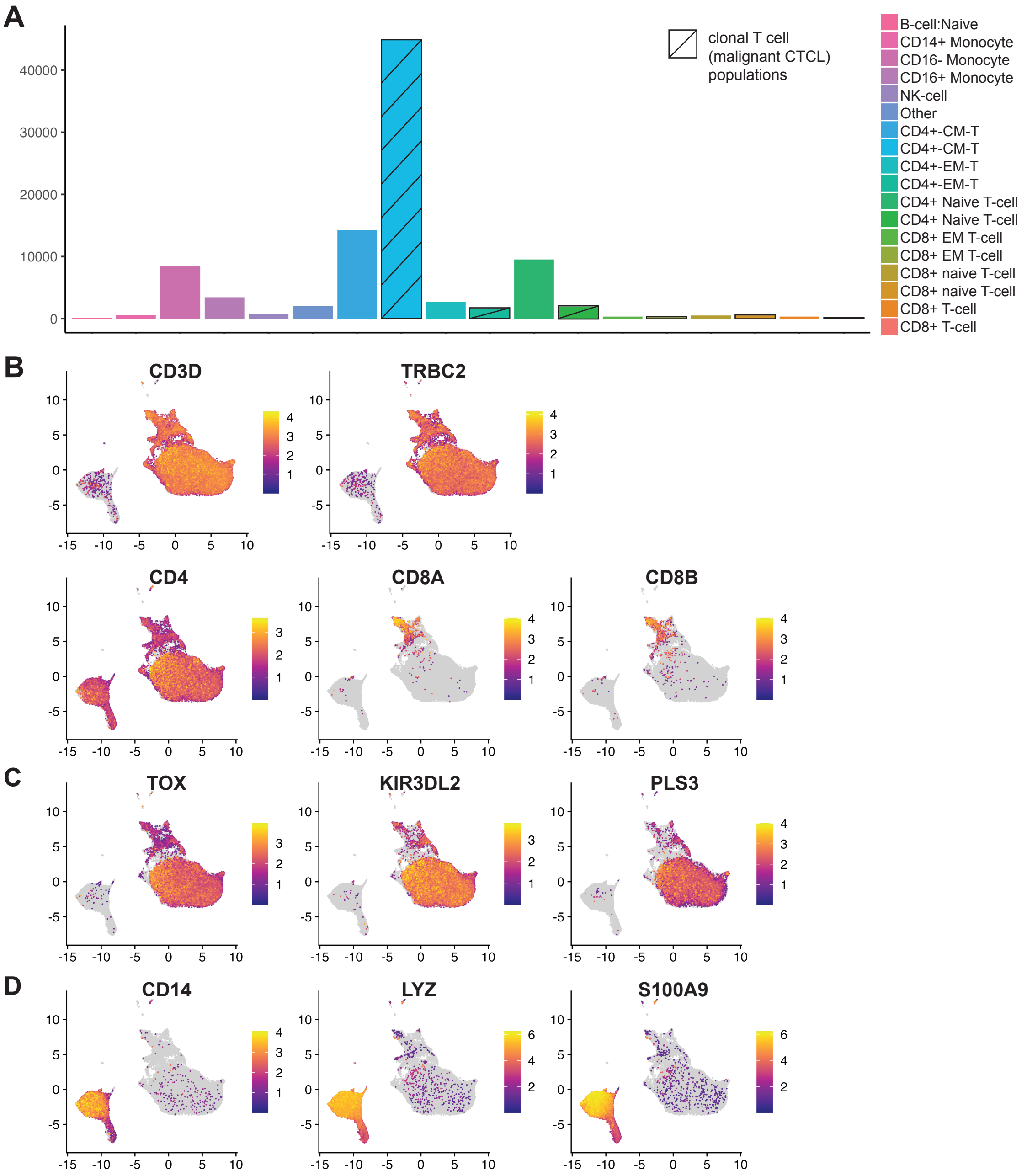

Supplementary Figure 1. scRNAseq cell type distribution and canonical marker genes demonstrates separation of benign and malignant T cells.

(**A**) Distribution of cell types based on HPCA cell annotation analysis for 92,496 mononuclear cells from 16 PBMC samples from 6 CTCL patients. (**B-D**) UMAP projection all cells from (**A**) shows expression of selected canonical T-cell (**B**), CTCL (**C**), and myeloid (**D**) genes.

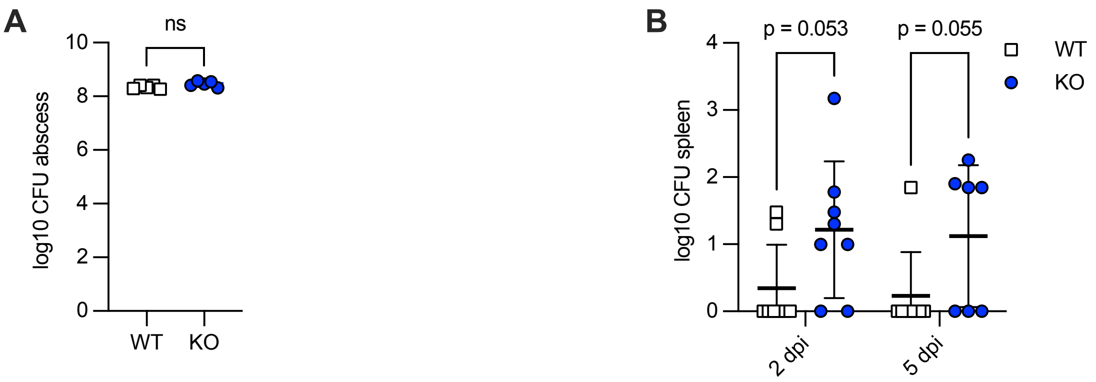

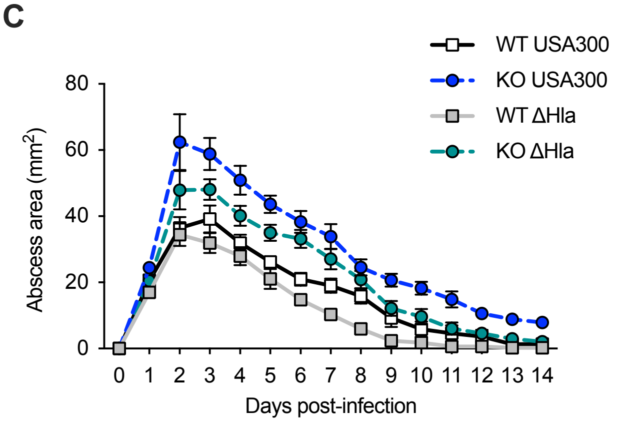

D

Abscess Three-way ANOVA			
	**Time**	**Genotype**	***S. aureus* strain**
**Time**	<0.0001	<0.0001	0.3324
**Genotype**		<0.0001	0.3849
***S. aureus* strain**			0.0008
three-way interaction term: p = 0.5414			

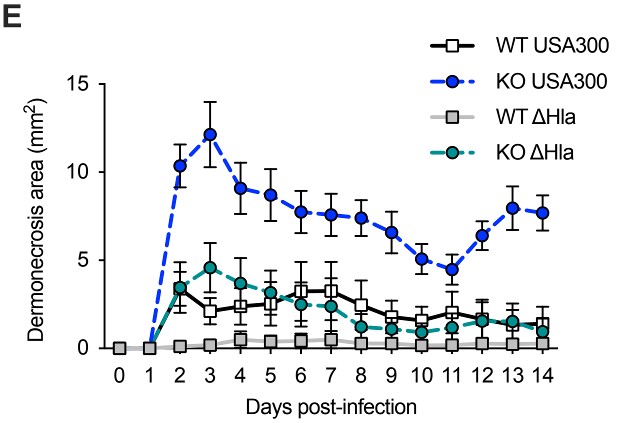

F

Dermonecrosis Three-way ANOVA			
	**Time**	**Genotype**	***S. aureus* strain**
**Time**	<0.0001	<0.0001	<0.0001
**Genotype**		<0.0001	**0.0249**
***S. aureus* strain**			<0.0001
three-way interaction term: p = 0.1333			

Supplementary Figure 2. Increase in abscess and dermonecrosis size in *S. aureus* skin infection in *Lair1* KO is maintained in the absence of Hla.
(**A**-**B**) WT and *Lair1* KO mice were infected subcutaneously with $1x10^7$ CFU *S. aureus* USA300 LAC. Bacterial growth was quantified as CFU recovered from homogenized skin punch biopsies collected at 2 days post-infection (dpi) (**A**) and from spleen at 2 dpi and 5 dpi (**B**). Statistical analysis for panels (**A**-**B**) by unpaired t-test. Significance: ns, not significant. (**C**-**F**) WT and *Lair1* KO mice were infected on opposite flanks with USA300/LAC and isogenic Hla knock-out strain (ΔHla), then lesion size measured over 14 days. Abscess area is plotted over 14 days (**C**) and shown with corresponding hypothesis testing (**D**) shown above; dermonecrosis area is plotted over 14 days (**E**) and shown with corresponding hypothesis testing (**F**). Three-way ANOVA tables show p-values for single variable effects shown on the diagonal, two-way interaction effects shown between respective variables, and three-way interaction effect specified below (**D**, **F**), with significant interaction terms involving genotype bolded.

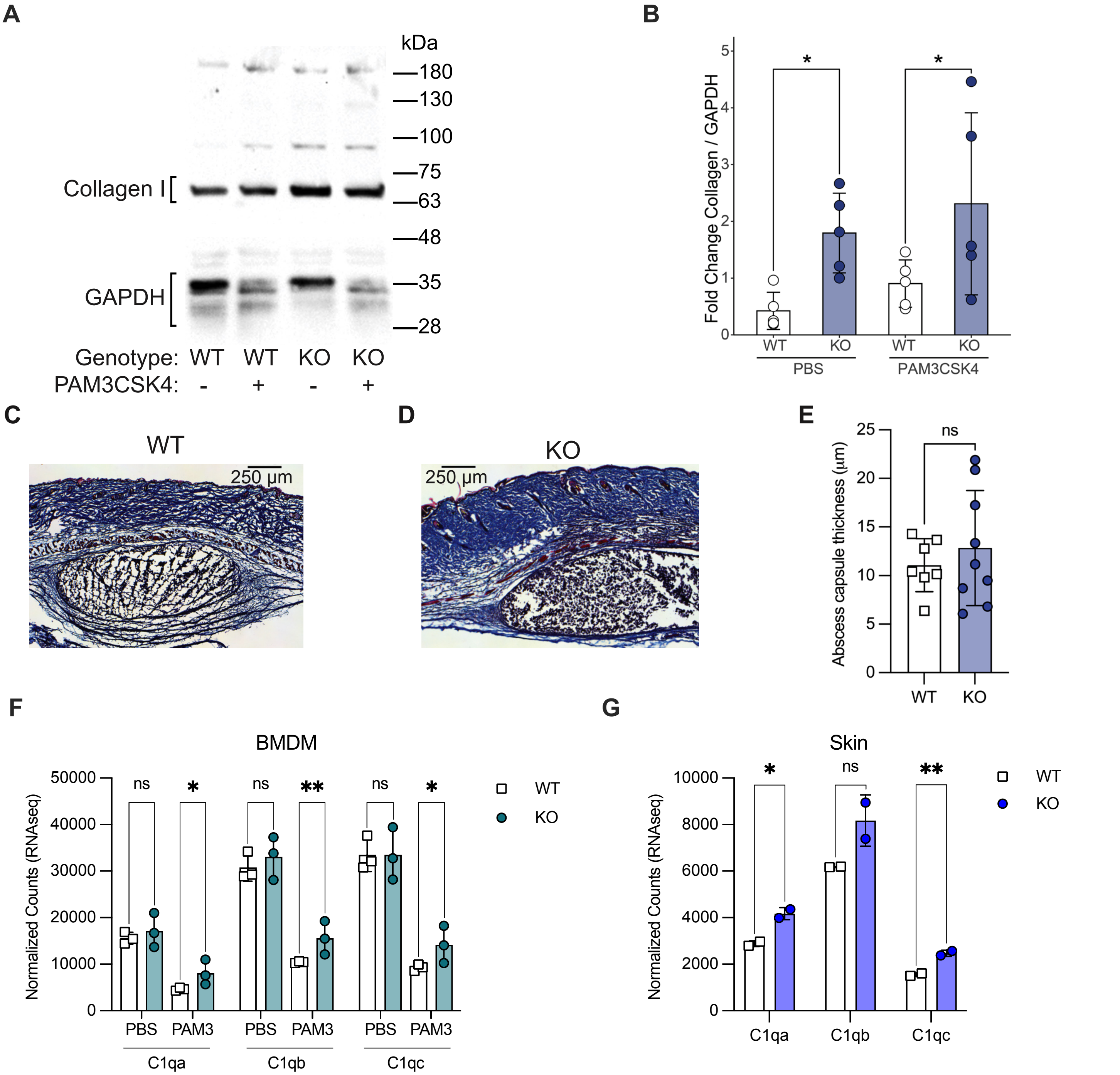

Supplementary Figure 3. Higher collagen protein and complement C1q expression in *Lair1* KO *S. aureus*-infected skin abscess and macrophages.

(**A**) Western blot demonstrating representative Collagen I and GAPDH staining from BMDMs treated with PBS or PAM3CSK4. (**B**) Quantification of Western blot band intensity in WT and KO BMDMs representative of 3 independent experiments. Statistical analysis by two-way ANOVA with multiple t-tests. Significance: *, $p < 0.05$. (**C-D**) Example images from trichrome stained SSTI skin lesions at 1 dpi from WT (**C**) and *Lair1* KO (**D**) are shown, with abscess marked with an asterisk (dermonecrosis not yet developed at 1 dpi), as well as average capsule thickness at 3 points per lesion, measured in 4 WT and 5 KO lesions across multiple sections (**E**). Statistical analysis by unpaired t-test. Significance: ns, not significant. (**F-G**) Normalized gene counts (RNAseq) for *C1qa*, *C1qb*, and *C1qc* in WT and *Lair1* KO BMDMs stimulated with PBS or PAM3CSK4 (PAM3) (**F**) and in *S. aureus*-infected skin (**G**). Statistical analysis for panels (**F-G**) by DESeq2 adjusted p-value. Significance: ns, not significant; *, $p < 0.05$; **, $p<0.01$.

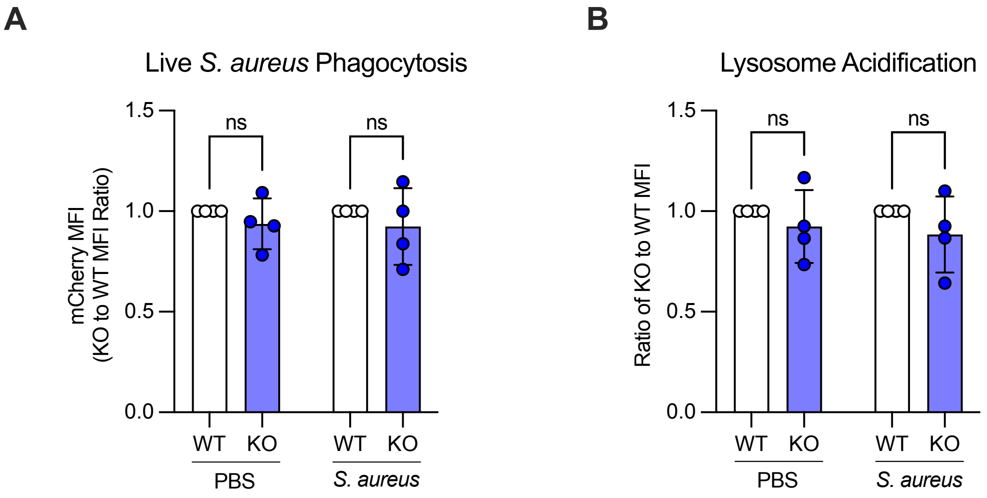

Supplementary Figure 4. *Lair1* KO and WT monocytes exhibit equivalent phagocytosis of *S. aureus*. Classical monocytes were isolated from WT and *Lair1* KO bone marrow. (**A**) Monocytes were incubated for 30 minutes with PBS or mCherry-expressing *S. aureus* at MOI 50:1 and subjected to flow cytometry. mCherry MFI is shown as a ratio of KO to WT fluorescence. (**B**) Ratio of WT to KO lysosome probe MFI measured by flow cytometry indicates lysosome acidification in monocytes treated with PBS or *S. aureus* at MOI 100:1 for 30 minutes. Statistical analysis by two-way ANOVA with Fisher's LSD. Significance: ns, not significant

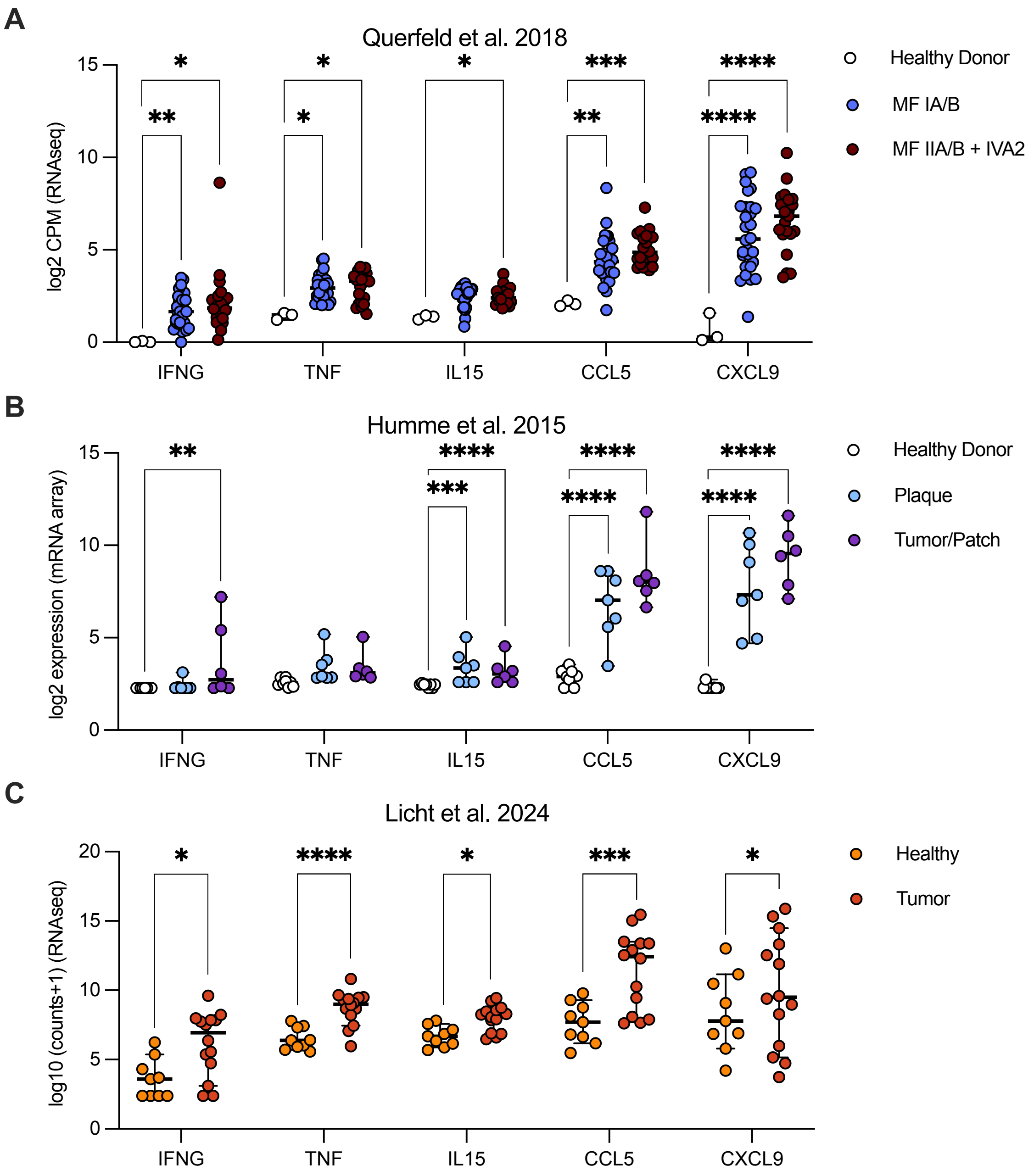

Supplementary Figure 5. Some cytokines elevated in CTCL are not elevated in *Lair1* KO.
Publicly available CTCL skin biopsy RNA-seq from Querfeld et al. 2018 (7) (**A**), Humme et al. 2013 (48) (**B**), and Licht et al. 2024 (49) (**C**) were analyzed using DEseq2. Normalized expression for IFNG, TNF, IL15, CCL5, and CXCL9 is shown. Statistical analysis for all panels by DEseq adjusted p-value. Significance: *, p<0.05; **, p<0.01; ***, p<0.001; ****, p<0.0001.

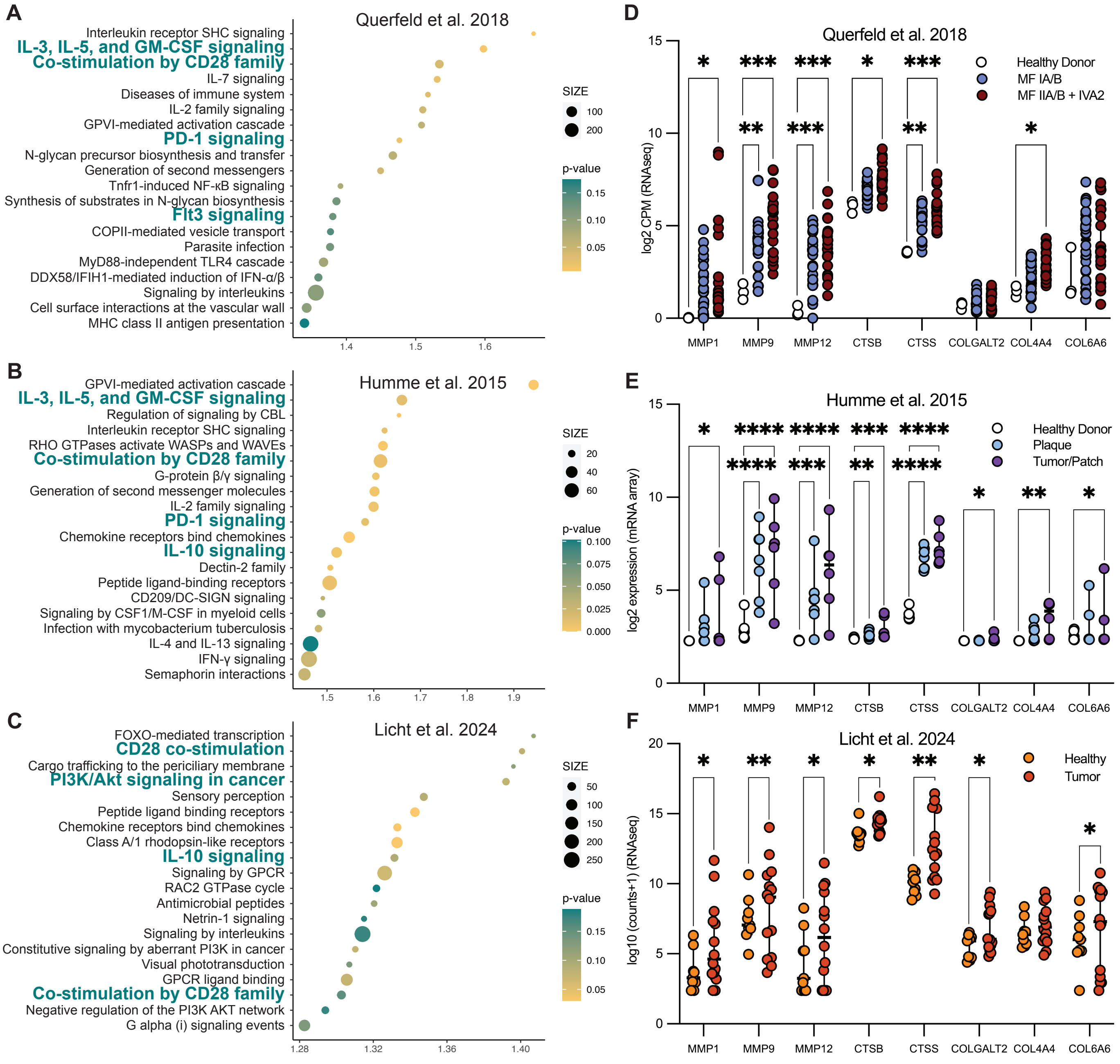

Supplementary Figure 6. Several collagen and ECM remodeling genes are elevated in CTCL. Publicly available CTCL skin biopsy RNA-seq from Querfeld et al. 2018 (7) (**A**, **D**), Humme et al. 2013 (48) (**B**, **E**), and Licht et al. 2024 (49) (**C**, **F**) were analyzed using DEseq2 and GSEA. (**A-C**) Top twenty pathways elevated in CTCL by GSEA human reactome analysis are shown with GSEA-defined enrichment scores and nominal p-value. (**D-F**) Normalized expression is shown for collagen pathway genes significantly increased in at least two datasets, including *MMP1*, *MMP9*, *MMP12*, *CTSB*, *CTSS*, *COLGALT2*, *COL4A4*, and *COL6A6*. Statistical analysis for panels (**D-F**) by DEseq adjusted p-value. Significance: *, p<0.05; **, p<0.01; ***, p<0.001; ****, p<0.0001.
