# Supplementary material for: LAIR1 prevents excess inflammatory tissue damage in Staphylococcus aureus skin infection and cutaneous T cell lymphoma
Source: JCI Insight. 2025 Nov 13;11(1):e183935. doi: 10.1172/jci.insight.183935 (PMC12890506; doi:10.1172/jci.insight.183935)
Supplement: Unedited blot and gel images [file jciinsight-11-183935-s250.pdf]

UNEDITED WESTERN BLOT IMAGES

Dorando et al. ms# 183935

COLLAGEN I

A

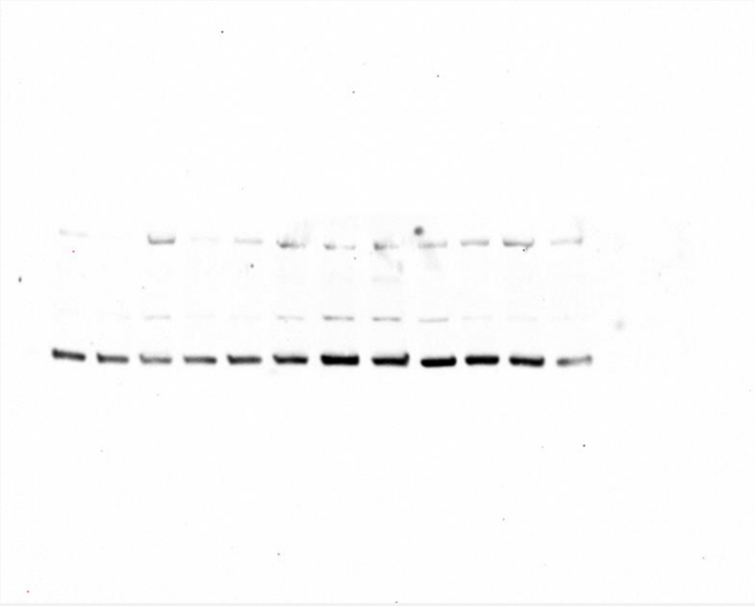

B

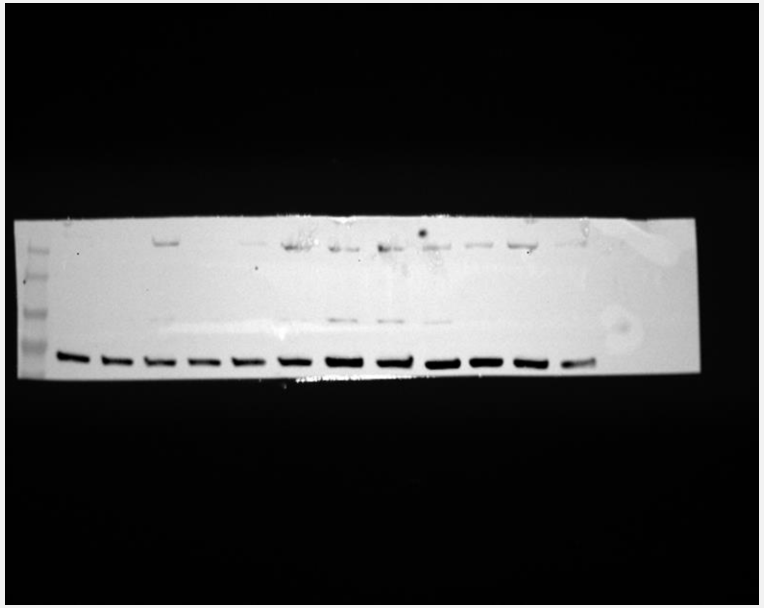

(A) (White background) Unedited image produced and analyzed by Invitrogen iBright 1500 and included in Figure S3
(B) (Black background) Unedited image with ladder and cut visible

GAPDH

A

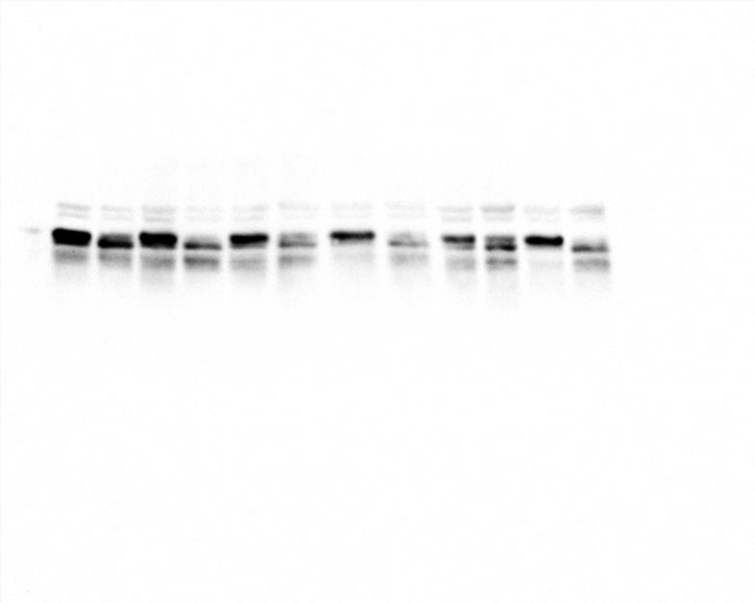

B

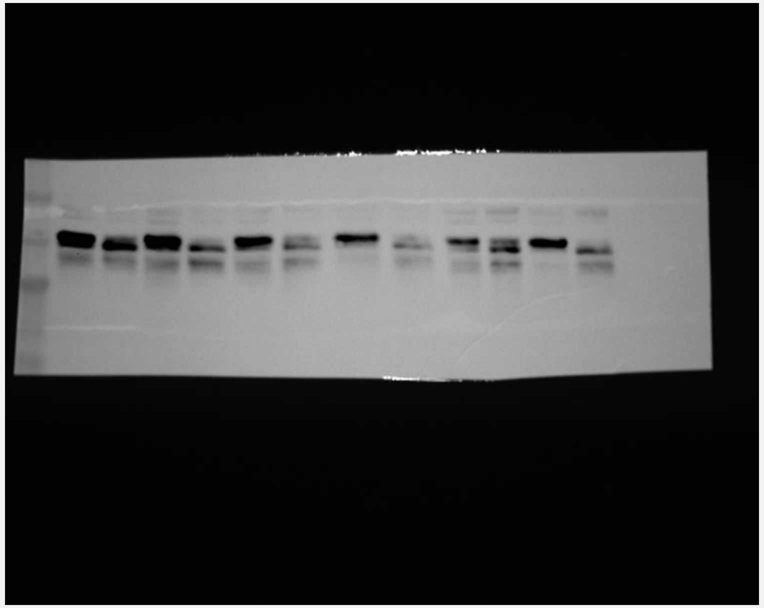

(A) (White background) Unedited image produced and analyzed by Invitrogen iBright 1500 and included in Figure S3
(B) (Black background) Unedited image with ladder and cut visible
